# Supplementary material for: The genomic footprint of whaling and isolation in fin whale populations
Source: Nat Commun. 2023 Sep 12;14:5465. doi: 10.1038/s41467-023-40052-z (PMC10497599; doi:10.1038/s41467-023-40052-z)
Supplement: Supplementary file 1 — Supplementary Information [file 41467_2023_40052_MOESM1_ESM.pdf]

Supplementary information for the manuscript by Nigenda-Morales and Lin et al., 2023. "The genomic footprint of whaling and isolation in fin whale populations"

Supplemental Methods

Genotyping, heterozygosity and ROH using a fin whale reference genome

In population genomic studies, using the genome from a species that is not the focal species as reference could potentially be problematic if both species diverged long ago because they have accumulated genetic variation independently and might not be useful to detect variation in the other species. To determine if using the minke whale genome as reference in our fin whale dataset would cause a substantial difference in genotyping statistics and in genomic diversity estimations (i.e. genome-wide diversity and runs of homozygosity (ROH)), we used a fin whale genome that has been made recently available (GCA_023338255.1). However, its annotation is not publicly available, preventing us from using it as the primary reference genome in this study. We randomly selected a subset of 10 fin whale samples [5 per population (ENP: ENPAK24, ENPAK30, ENPCA04, ENPCA08 and ENPWA15; GOC: GOC006, GOC050, GOC071, GOC086, GOC100)] to perform these analyses (we named this dataset 10-fin-ref). First, we performed all the steps of our genotyping pipeline using the fin whale genome GCA_023338255.1, except the snpEff and SIFT annotation step that requires a genome annotation. Second, with the genotyping data obtained using the fin whale reference genome, we calculated the genome-wide heterozygosity for the 10 individuals as previously mentioned in the main manuscript and compare them with the results obtained with the data using the minke whale reference genome. We performed a two-tailed Wilcoxon test to determine if the differences observed were statistically significant due to the reference genome choice. Finally, we calculated the total ROH length with the bcftools software and compared them with the results obtained using the minke whale genome as reference and performed a two-tailed Wilcoxon test.

Grid search for the ENP population 3-epoch model

The time and magnitude of a population contraction are difficult to disentangle in demographic models[1,2]. To account for this correlation of dip in current population size (N_{CUR}) and time of most recent contraction (T_{CUR}), potentially caused by whaling, we carried out two grid searches exploring the full likelihood surface of the 3Epoch model for the ENP population using ∂a∂i. First, we did a broad grid search aimed to find the range of possible parameter pairs that are within two log-likelihood units of the maximum likelihood estimate (MLE) for the ∂a∂i 3-epoch model. Based on the parameter values estimated from empirical data for this model in ∂a∂i units, we fixed the population size in the previous epoch (nuB = 1.4511; nuB is the N_{BOT} equivalent in ∂a∂i units) and the total time of two recent epochs (TB+TF = 0.1342; TB and TF are the T_{BOT} and T_{CUR} equivalent in ∂a∂i units respectively). Then, we spaced 100 grid points for current population size (nuF = 0.0001 ~ 2; nuF is the N_{CUR} equivalent) and time of most recent

contraction (*TF* = 1e-5 ~ 0.134) on a log-10 scale. We obtained the expected SFS for each *nuF-TF* pair and calculated the multinomial log-likelihood relative to empirical SFS as described in ∂a∂i inference methods. For easier interpretation, the corresponding values of N_{CUR} (current effective population size; $N_{CUR} = nuF*N_{ANC}$) and T_{CUR} (time of most recent population size change; $T_{CUR} = TF*2*N_{ANC}$) are calculated using the best-fit N_{ANC} reported in Table S7. Second, to further explore the fine-scale likelihood surface within two log-likelihood units of the maximum likelihood estimate (MLE) of the broad grid search, we conducted an additional zoomed-in grid search with *nuF* ranged from 0.0030 to 0.3651 and *TF* ranged from 1e-5 to 0.0016, i.e. the range of possible parameter pairs within two log-likelihood units of the MLE reported above, using 400*400 grid points on a log-10 scale. To shorten computation time, the grid points falling outside of the ridge region (black box in Fig. S14B) were dropped in the computation of expected SFS, resulting in a total of 27738 computed grid points.

Demographic inference for the ENP population using the genotype-filter-free dataset

Stringent genotype filtering could cause a bias against rare alleles. Therefore, we generated an additional variants dataset for the ENP individuals without any genotype-level filters or the 20% missingness filter applied after genotype filtration, which we designated as the "genotype-filter-free" dataset. This dataset was then used to estimate a putatively neutral SFS under which we ran confirmatory demographic inferences for this population.

After generating the vcf file using the genotype-filter-free dataset, we followed the same procedure to identify neutral regions and 418,757,124 sites were defined as such. We followed the same procedure to generate the new filter-free SFS as for the filtered dataset (main result), where the same projected haplotype size (N=44) was used. We built this additional filter-free neutral SFS for the ENP population to test if our demographic estimates calculated with the filtered dataset (main results) are bias against rare alleles, which the results show they are not. Our demographic inferences under this filter-free SFS followed almost the same procedure described for the filtered dataset (main results), the only difference was that in addition to testing the 1Epoch through 4Epoch models together with the 3EpochTcur2 and 3EpochTcur3 models we also included a 3EpochTcur1 model (Table S9), which has the recent bottleneck time fixed to one generation. We performed the demographic inferences with ∂a∂i and fastsimcoal2.

Comparison of baleen whales genome assemblies

We used BUSCO v.4[3,4] to summarize the genome assembly and annotation qualities for Balaenopteridae species available in 2021. The minke whale assembly used has the second highest annotation completeness among six assemblies compared (Table S16; Fig. S21), further confirming that this reference genome choice is unlikely to impact downstream analyses. Given the high quality and availability of baleen genome assemblies, our study does not generate a new reference genome but rather focuses on filling the gaps for population-level high quality whole genome resequencing datasets in baleen whales.

Supplemental Results

Comparing genotyping and diversity estimates using the minke and fin whale reference genomes

There were no great differences in genotyping or genetic diversity statistics when we used the minke whale or fin whale genome as the reference (Table S2). For example, the average mapping rate to both genomes were higher than 99% in both cases (minke whale = 99.09%, fin whale = 99.49%) with the fin whale genome having a slightly higher mapping rate, as expected. Although the average number of heterozygous sites identified was slightly higher when aligned to the fin whale genome (Table S2; minke whale = 1,290,413, fin whale = 1,457,881), which could be interpreted as obtaining higher genetic diversity, we observed a slightly higher genome-wide diversity when the minke whale genome but not the fin whale genome is used as reference (Fig. S1A-B). This is because more sites were called when the fin whale genome is the reference, as expected (Average number of called sites; Table S2), resulting in a lower genome-wide number of heterozygous sites per called sites. Therefore, we do not observe a slight underestimation of diversity using the minke whale genome reference, if anything, we have a slight overestimation of diversity. This overestimation is minimal when we calculate it for the 10 individuals we reanalyzed, with an average heterozygosity rate of 0.00140 (when mapping to fin whale) vs 0.00142 (when mapping to the minke whale), an increase of less than 1.5%. Therefore, the bias in heterozygosity estimates is negligible to our main results. Additionally, no significant differences between the two reference genomes were found in genome-wide heterozygosity (Figs. S1A, S1B) or total ROH length (Figs. S1C, S1D). All these results indicate that regardless of the reference genome used, the genotyping pipeline results and the genomic diversity estimates are similar, suggesting that using the minke whale genome as a reference does not significantly affect the results of our analyses.

Grid search

The broad grid search revealed a ridge of log-likelihood within two units of MLE derived from the parameter pairs, with *nuF* ranging from 0.0030 to 0.3651 (corresponding N_{CUR}: 49 ~ 6018 individuals) and *TF* from 1.0e-5 to 0.0016 (corresponding T_{CUR}: 0.33 ~ 53.44 generations; Fig. S14A). This ridge of likelihood suggests that compared with a very recent contraction event potentially caused by whaling, a more ancient contraction event could fit the data similarly well. However, we note that, only three out of the 10,000 parameter pairs resulted in a better likelihood (MLE log-likelihood: -187.3507) compared with the parameter pairs inferred from the empirical SFS (log-likelihood: -187.4310; Table S5), all of which are parameter pairs describing recent and strong contraction events (Fig. S14B). In the zoomed-in grid search, we found that there is a consistent yet small (less than 0.1) increase in the log-likelihood favoring a more severe and recent contraction event within three generations, which corresponds to the more intense whaling period for this species between 1940 and 1980[5,6]. The MLE in the zoomed-in grid search reported a log-likelihood of -187.3377 at the parameter pair of *nuF* = 0.0034; *TF* = 1.11E-05. Similar to the broad grid search, the 173 out of 27,738 parameter pairs that have better likelihood than the parameter pairs inferred from the empirical SFS, all described a more recent and severe contraction (Fig. S14C).

Overall, these grid search analyses confirmed the correlation of current population size (N_{CUR}) and time of most recent contraction (T_{CUR}). We showed that the optimal log-likelihood inferred by the recent decline scenario has a minimal improvement over more ancient contraction scenarios but also caution that the ancient contraction scenarios could fit the data equally well.

Gulf of California population model selection

The likelihood ratio test (LRT) and Akaike information criterion (AIC) results for the Gulf of California single-population models show that for both demographic inference methods, ∂a∂i and fastsimcoal2, the 4-epoch model has the maximum likelihood estimate (MLE) and is significantly better than all the other models (Table S5). However, the parameter estimates obtained for this model are not consistent between methods, lack convergence and have large confidence intervals (Tables S5, S7), which could indicate overfitting of this model. The same conclusion can be reached for the 3-epoch model. Also, when visually comparing the fit of the SFS from all the models for this population to the SFS of the data, none of them show a close fit (Fig. S13B). These results could be explained by the lack of information about gene flow in single-population models[1,7], which although highly limited, gene flow seems to have played a very important role during the evolutionary history of the Gulf of California population. Therefore, we decided to infer the demographic history of the GOC population based on our analysis of the joint SFS of two populations, which has been previously shown to contain more information than single-population SFS, specifically information about past gene flow[1].

Two population model selection

Two models (AncestralSizeChange-Split-Isolation-AsymmetricMigration and AncestralSizeChange-Split-AsymmetricMigration-GOCChange) had a higher log-likelihood than the chosen model (AncestralSizeChange-Split-AsymmetricMigration).

We first ruled out the model of ancestral size change with asymmetric migration and size change in the Gulf population (AncestralSizeChange-Split-AsymmetricMigration-GOCChange) due to its non-significant improvement over the AncestralSizeChange-Split-AsymmetricMigration model in the LRT for the fastsimcoal2 estimation (Table S12, see model selection criteria 5 in Methods section), large confidence intervals (Table S11, model selection criteria 6) and lack of convergence (Table S5, model selection criteria 4). However, we acknowledge that ∂a∂i favored this model and some of the parameters, particularly the divergence time between ENP and GOC populations are similar to the one reported in ref.[8].

Then, although the results for both demographic inference methods show that the model of ancestral size change with isolation followed by asymmetric migration (AncestralSizeChange-Split-Isolation-AsymmetricMigration) has significantly better log-likelihood than the rest of the nested models (Table S12), the log-likelihood lacked good convergence (Table S5, model selection criteria 4), the parameters estimated for this model, are not entirely consistent between methods (Table S11, model selection criteria 3), or have large confidence intervals (Table S11, model selection criteria 6). These features could indicate an overfitting of this model even when it shows better log-likelihood. Therefore, we chose the model describing an ancestral size change event before population divergence and sustained asymmetric migration between populations (AncestralSizeChange-Split-AsymmetricMigration) as the model that best represent our data.

This model has the third highest likelihood (Tables S5, S12), narrow confidence intervals (Table S11), good parameter convergence (Table S5) and good fit to the data (Fig. S17).

Theoretical expectation for diversity loss

To aid readers interpret the simulation output and understand the theoretical basis of diversity loss in small populations, we provide a theoretical calculation on diversity decline in the GOC population if no migration is allowed and in the ENP population with various recovery scenarios.

To emphasize that existing migration with ENP, although low, is essential for sustaining the GOC population, we provide a theoretical calculation on diversity decline in the GOC population using the demographic scenarios inferred in Fig. 4B except that no migration is allowed. Using the classic mutation-drift model (Hamilton p. 192, Eq. 5.37), the expected autozygosity at generation t (F_t) is determined by the expected autozygosity at previous generation (F_{t-1}), the effective population size (N_e) and the per-generation mutation rate (μ). At each generation, the expected heterozygosity (H_t) is determined by $H_t = 1 - F_t$.

$$F_t = \frac{1}{2N_e}(1-\mu)^2 + (1 - \frac{1}{2N_e})(1-\mu)^2 F_{t-1}$$

We used the average empirical observed heterozygosity for the ENP population (Fig. 2B) as a proxy for expected heterozygosity for the GOC population at the time of divergence: $H_{t=0}$ = 1.76E-03 (heterozygotes/bp), therefore $F_{t=0}$ = 1-1.76E-03. We set N_e = 114, μ = 2.77E-08 mutations/bp/generation, T = 616 generations from the demographic inference results and calculated $H_{t=616}$ recursively. The theoretically derived current heterozygosity in the GOC is $H_{t=616}$ = 1.29E-04. This value is similar to the mean simulation derived current heterozygosity (H = 2.02E-04) in the GOC if no migration is allowed. We demonstrated that the current empirical heterozygosity level in the GOC population (H = 1.13E-03, Fig. 2B) is five to ten-fold higher than the assumed no migration scenario using both theory and simulations, suggesting the importance of low levels of migration in maintaining viability in the GOC population over its long period of isolation.

Similarly, we can also calculate the expected diversity decline for the ENP population and illustrate that the short number of generations passed from bottleneck is essential for the lagging impact on genetic diversity. The whaling bottleneck lasted for approximately three generations from empirical records (ie. ~70 years of whaling / 25.9 years per generation = 2.7 generations) and one to two generations from demographic inference (see main text Results, Demographic reconstruction of whaling, divergence and gene flow). Using N_e = 305, we can derive from the equation above that $H_{t=2}$ = 0.9967*$H_{t=0}$. Therefore, only 0.33% heterozygosity has been lost during two generations of reduction.

Supplemental Discussion

Potential biases when using a reference genome from a closely related species for variant calling.

The potential biases of using a reference genome from a closely related species instead of the focal species include a lack of synteny, the intraspecific polymorphism might be underestimated due to the difference between the divergence at the population level and the divergence to the reference taxa and the definition of derived vs reference alleles may be problematic. However, these potential biases do not seem to affect our results when using the minke whale genome as reference. Synteny across large regions of the genome is not needed for the analysis of coding regions and might not be expected to play an important role when the mapping rate is high (99.09% reads mapped using minke whale) and the focus is on analyzing SNPs, which is the case in our study. Although our ROH analysis might be affected by a lack of synteny, given that we did not observe significant differences in total ROH length between reference genomes (Fig. S1C, D; minke whale vs fin whale), it seems synteny is not a problem in our study. Even if intraspecific polymorphism could be slightly underestimated when we use the minke whale genome as a reference, we do not observe such underestimation, if anything we observe a slight overestimation due to less accurate mapping, but this does not seem to affect the estimation of genome-wide heterozygosity and ROH as shown in our analyses comparing these diversity estimates between reference genomes (Fig. S1A-D). Additionally, because the divergence time between the minke whale and the two fin whale populations is approximately the same, using its genome to identify the ancestral and reference alleles is not expected to introduce a significant bias.

The BUSCO statistics for the fin whale assembly that has been published[9] are worse than the minke whale assembly that we used. The BUSCO statistics for the fin whale genome are as follow: BUSCO Cetartiodactyla C:83.4%[S:82.1%, D:1.3%], F:4.1%, M:12.5%, N50 = 2.49xE07, L50 = 27, 17,307 genes[9]. Whereas the BUSCO analysis reported in the NCBI website for the minke whale genome we used are: BUSCO Cetartiodactyla C:97.6%[S:96.3%, D:1.4%], F:1.1%,M:1.3%, N50 = 1.28xE07, L50 = 57, 26,806 genes (https://www.ncbi.nlm.nih.gov/data-hub/genome/GCF_000493695.1/). Therefore, even if such fin whale genome annotation was available, using an assembly of less quality could negatively impact our results, especially the accuracy of the deleterious variation analyses, and given the results of our reanalysis comparing genotyping and diversity estimates it seems that using the fin whale genome assembly and annotation will not make a significant difference for our main results and conclusions.

Gulf of California demographic history

The two-population model that was selected (AncestralSizeChange-Split-AsymmetricMigration) shows that the Gulf of California population was founded and diverged from the ENP population around 16 kya and has remained at a very small effective populations size (N_e) of around one hundred individuals since then (~114 or ~124 individuals estimated with ∂a∂i and fastsimcoal2, respectively). Previous estimations based on microsatellite and mitochondrial loci inferred the Gulf of California population divergence from the Eastern North Pacific population between 2.3[8] and 9.3[10] kya. These estimates might be too recent to generate some of the genomic diversity patterns that we observe in the GOC population. Rivera-León et

al. 2019[8] also estimated the contemporary and long-term N_e of the GOC population at 360 and 40 individuals, respectively. Their long-term N_e inference is somewhat similar to ours, however their long-term immigration estimate of 0.89 (95% HPDI: 0.040-3.13) migrants per generation from the Eastern North Pacific into the Gulf of California, has a considerable larger uncertainty and is more than double the 0.39 migrants/generation we estimated for both ∂a∂i (95% CI: 0.35 – 0.42) and fastsimcoal2 (95% CI: 0.32 – 0.40). Therefore, our results show that analyzing the joint SFS of the ENP and GOC populations derived from whole-genome sequencing data can provide better and more accurate estimations of the demographic history of the Gulf of California fin whale population. However, we acknowledge that the mtDNA-based estimations can differ from ours because this genetic marker is maternally inherited and non-recombining.

Detection of whaling bottleneck from SFS

In conservation biology, it is important to reliably infer the time and magnitude of past population bottlenecks of endangered species to implement effective management and recovery strategies. Analyzing the SFS of populations allow us to detect population reductions because a decrease in rare variants is expected after such event[11]. However, it is known that applying stringent filters on SNPs and genotypes to identify reliable genetic variants could excessively remove and artificially bias results against rare variants[12,13]. Therefore, in our study, there might be a concern that the whaling bottleneck we detected for the ENP population could be an artifact due to an overly stringent genotype filtering. To ease this concern, we redid our demographic inference using an SFS generated without applying any filters for genotyping calling (genotype-filter-free dataset). These results are very similar to our results based on the genotype calling filtered data, suggesting that the population reduction that overlaps with the whaling period is not due to a methodological artifact introduced during the genotype calling filtering process.

Additionally, we would like to point out that **1)** it has been previously shown that at a relatively adequate sequencing coverage of 20x various genotype calling and filtering methods have negligible effects on the shape of the SFS[12,13], and the mean coverage per sample in our study was 27.01x (Table S1). Our results are consistent with these previous findings that no significant change in SFS shape is expected for our high-coverage data under different filtering schemes. **2)** The genotype filters we applied are not more stringent than the standard practice for filtering whole genome resequencing datasets used to infer the recent demographic history in other endangered species[14–18]. **3)** Although the deficiency of rare alleles in SFS is an intuitive signature of bottlenecks, how to define this “deficiency” is still largely unclear. Depending on the demographic events occurring prior or after the bottleneck and its severity, the shape of SFS from bottlenecked populations may not be restricted to deficiency of rare alleles[11]. For example, in our study, the Gulf of California population had an even lower proportion of rare alleles (Fig. S13) yet did not experience a recent bottleneck event as the ENP population. **4)** SFS-based demographic inference methods, (e.g., ∂a∂i or fastsimcoal2) consider not only the rare alleles (i.e. singletons) but the full SFS spectrum to infer the best fit models[19]. **5)** The genotype likelihood-based approach, which is very powerful when applied to low or medium coverage sequencing data, it is not the most suitable for higher coverage dataset, like the one presented in our study (https://github.com/ANGSD/angsd/issues/131). **6)** All of our confirmatory tests suggest that we have the power to consistently detect the whaling bottleneck in the ENP population.

Ghost population models

Including a ghost population in the demographic analysis did not affect our migration estimates of less than one individual per generation into the GOC. However, the coincidence of the expansion observed in the selected ENP population model (3Epoch) and two-population model (AncestralSizeChange-Split-AsymmetricMigration) (Figs. 3A, 3C; Tables S7, S11) with the divergence time between the WNP and ENP populations estimated in the ghost population model ~4,300 generations ago (Table S14), could indicate that population structure existing at that time within the North Pacific might be confounded with size changes[20–24]. Testing this structure is not within the scope of our present work and further sampling will be necessary to resolve it. However, if this last scenario is true and the WNP and ENP have high migration rates among them, the demographic history depicted by our one-population ENP model could be representative of the entire North Pacific. This last result shows that considering ghost populations reveals the nuance of demographic reconstruction of whale populations[25–27].

Supplemental Figures

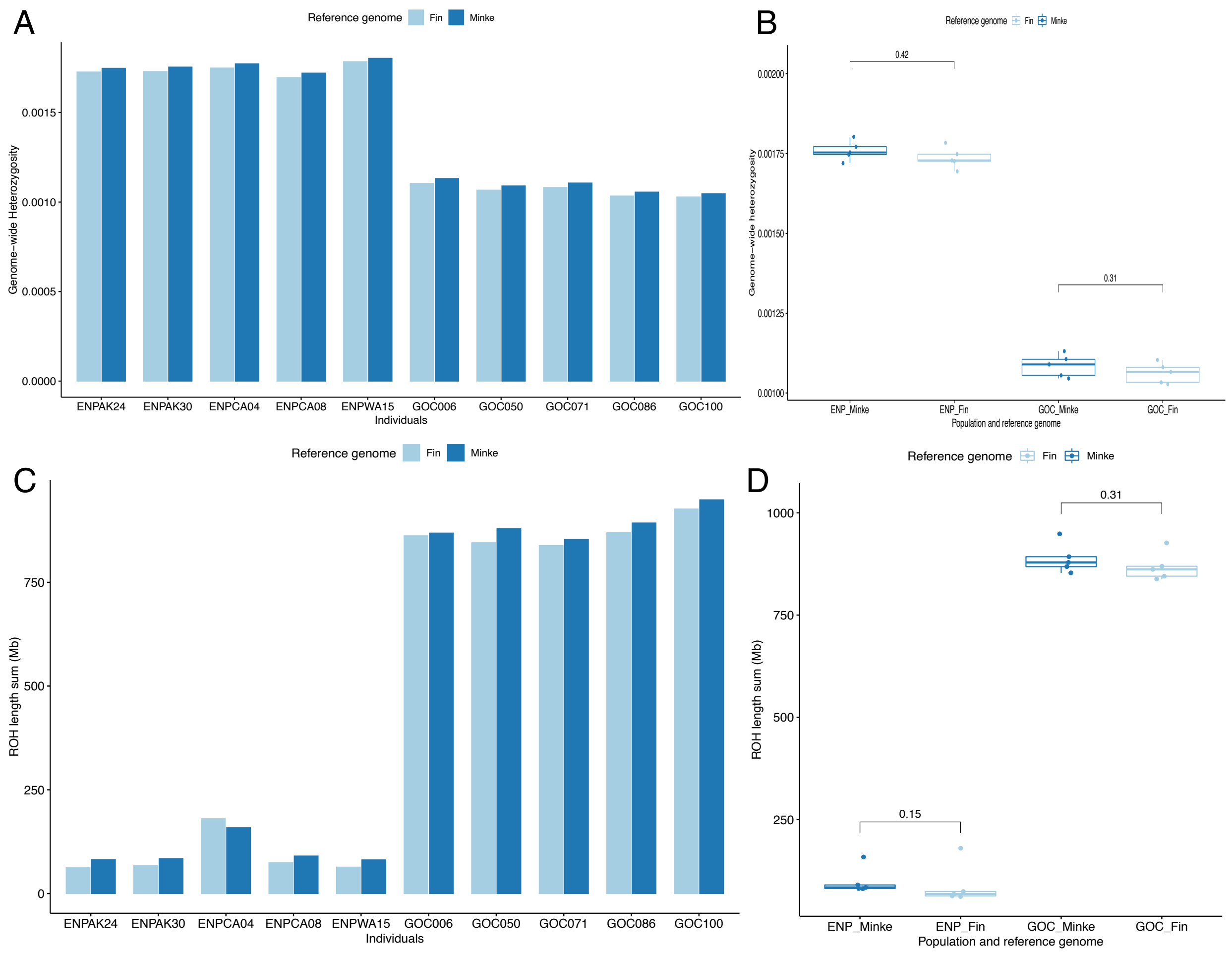

Fig. S1. Comparisons of the genomic diversity statistics using either the fin whale (GCA_023338255.1; light blue) or the minke whale (GCF_000493695.1; dark blue) reference genome. (A) The genome-wide heterozygosity for each individual using different reference genome. (B) Boxplot for the average genome-wide heterozygosity shown in (A). Within each population (ENP/GOC), we compared if the average genome-wide heterozygosity is significantly different by reference genome choice (Minke/Fin). P-value from the two-sided Wilcoxon test without multiple testing correction is annotated above. (C) The total length of ROH for each individual using different reference genome. (D) Boxplot for the total length of ROH shown in (C). For (B) and (D) the sample size is N=5 individuals per population, Gulf of California (GOC) and Eastern North Pacific (ENP). Within each population (ENP/GOC), we compared if the total length of ROH is significantly different by reference genome choice (Minke/Fin). P-value from the two-tailed Wilcoxon test without multiple testing correction is annotated above. For all boxplots (B and D), the notch indicates the median, and the boxes represent the 25th and 75th percentiles. The whiskers extend to data points no more than 1.5 * IQR (inter-quantile range) from the hinges and the points show the value for each individual.

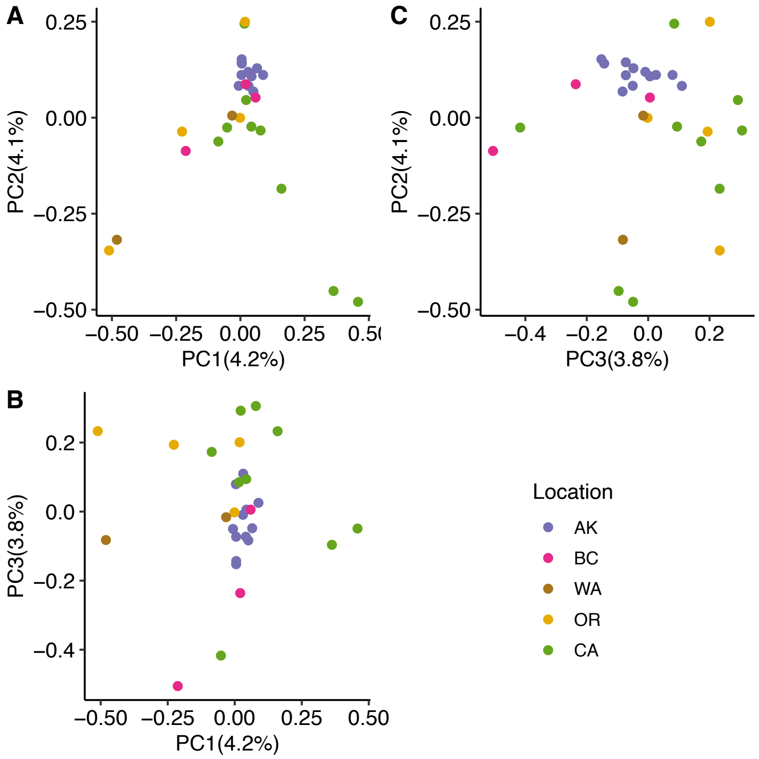

Fig. S2. Principal component analysis (PCA) within Eastern North Pacific samples. Additional PCA analyses were performed within ENP samples, excluding the Gulf of California samples. The first three PCs are plotted. **(A)** PC1 vs PC2, **(B)** PC1 vs PC3, **(C)** PC2 vs PC3. Samples are colored by sampling location (AK= Alaska, BC = British Columbia, WA = Washington, OR = Oregon, CA = California).

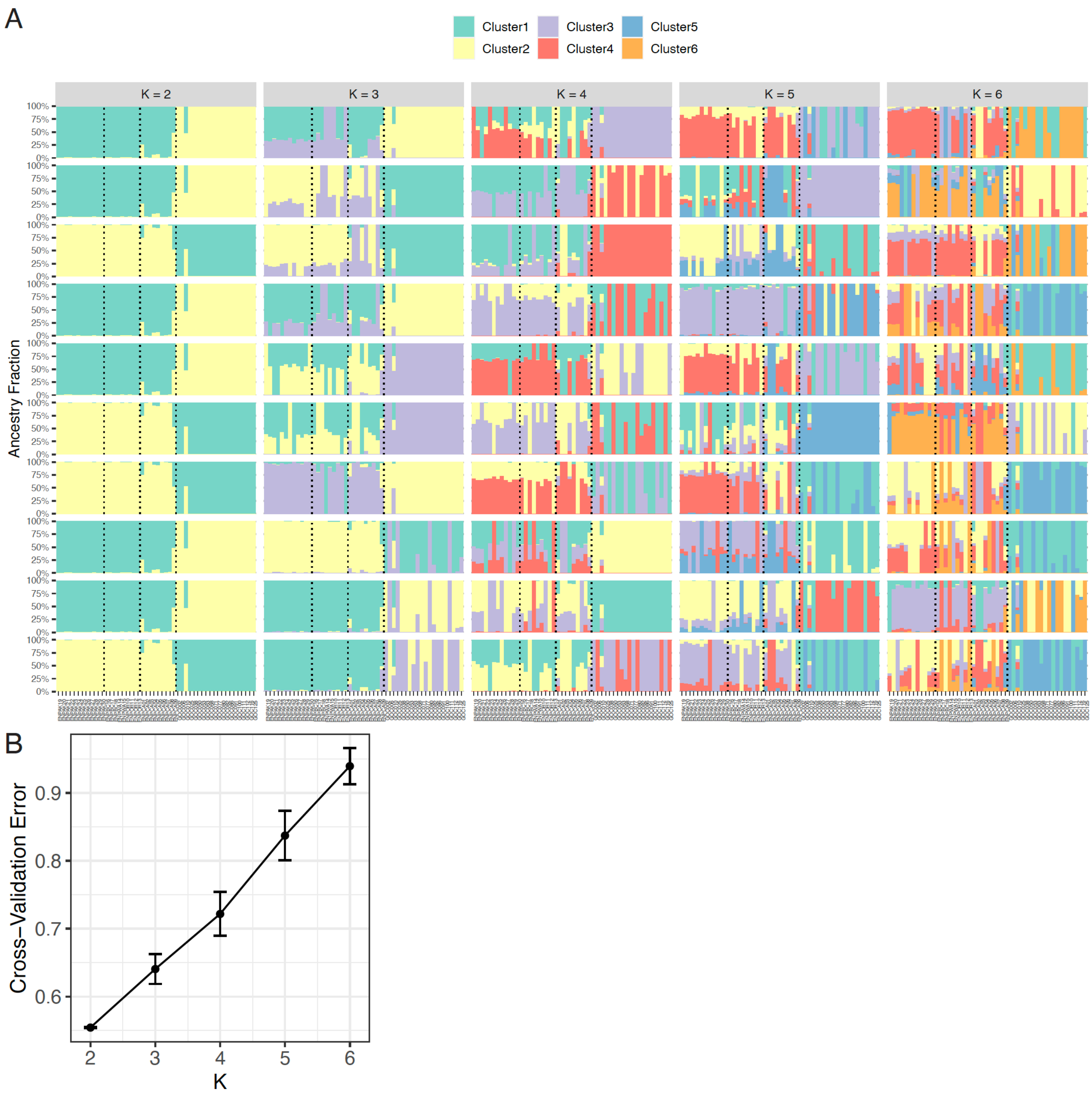

Fig. S3. Admixture plots. Admixture analyses with number of ancestral populations ranged from *K* = 2 to *K* = 6. **(A)** 10 random iterations of each *K* are presented. For this analysis the sample size was N=50 fin whales, 20 from the Gulf of California and 30 from the Eastern North Pacific. Individuals are ordered left to right by their sampling locations: Alaska (AK), British Columbia (BC), Washington (WA), Oregon (OR), California (CA) to the Gulf of California (GOC). The dashed lines separate Alaska, Middle ENP (BC, WA and OR), California and the Gulf of California individuals. **(B)** The Cross-Validation (CV) error from ten iterations per *K* is plotted, with points representing mean CV error values and error bars representing standard deviation. *K* = 2 had the lowest CV error.

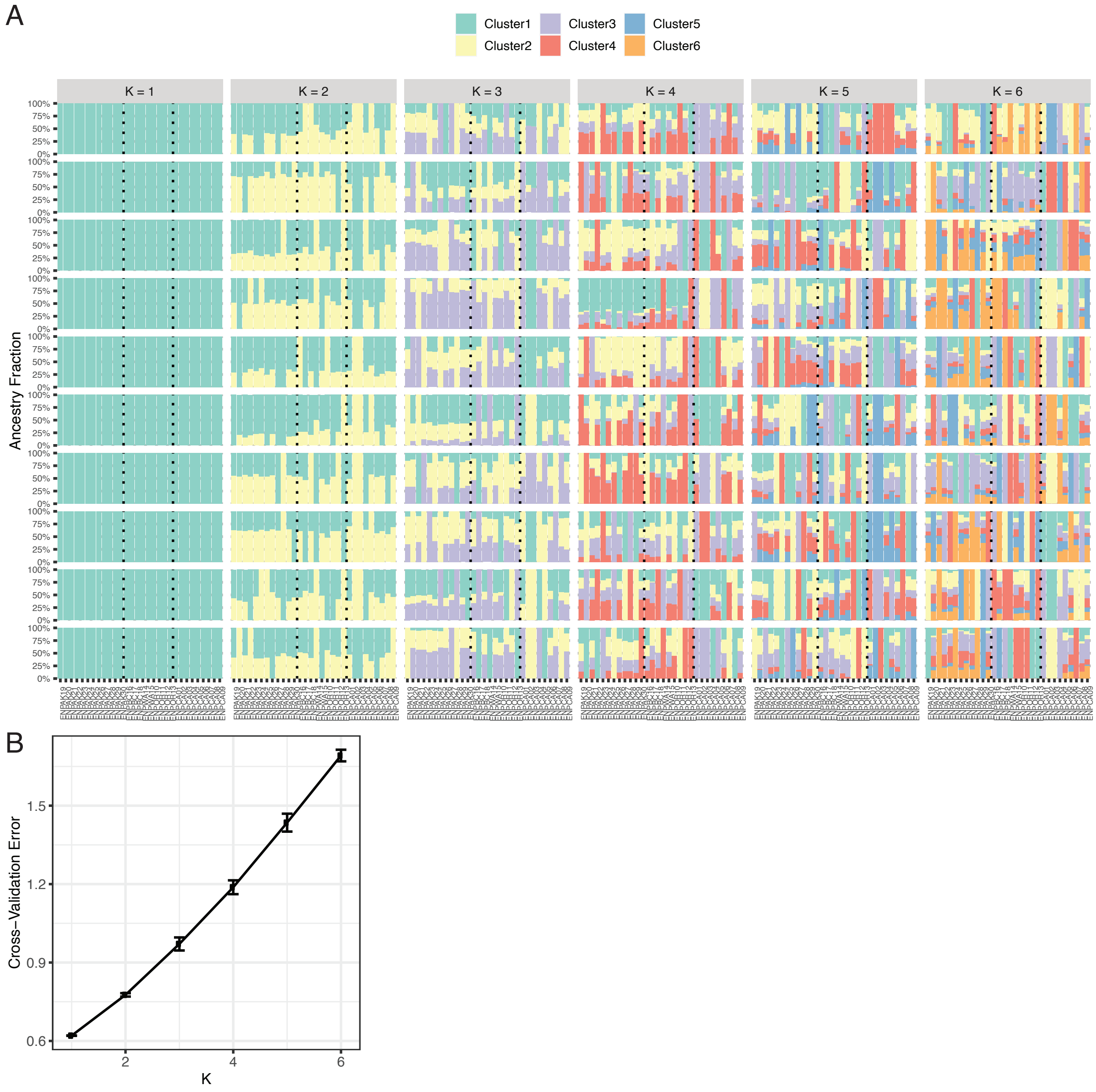

Fig. S4. Admixture plots within the Eastern North Pacific (ENP) individuals. Admixture analyses with number of ancestral populations ranged from $K = 1$ to $K = 6$. For this analysis the sample size was N=30 fin whales from the Eastern North Pacific. **(A)** Ten random iterations of each K are presented. Individuals are ordered left to right by their sampling locations: Alaska (AK), British Columbia (BC), Washington (WA), Oregon (OR) to California (CA). The dashed lines separate Alaska, Middle ENP (BC, WA and OR) and California individuals. No consistently reproducible ancestral proportion differences were found across individuals from these locations. **(B)** The Cross-Validation (CV) error from ten iterations per K is plotted, with points representing mean CV error values and error bars representing standard deviation. $K = 1$ had the lowest CV error.

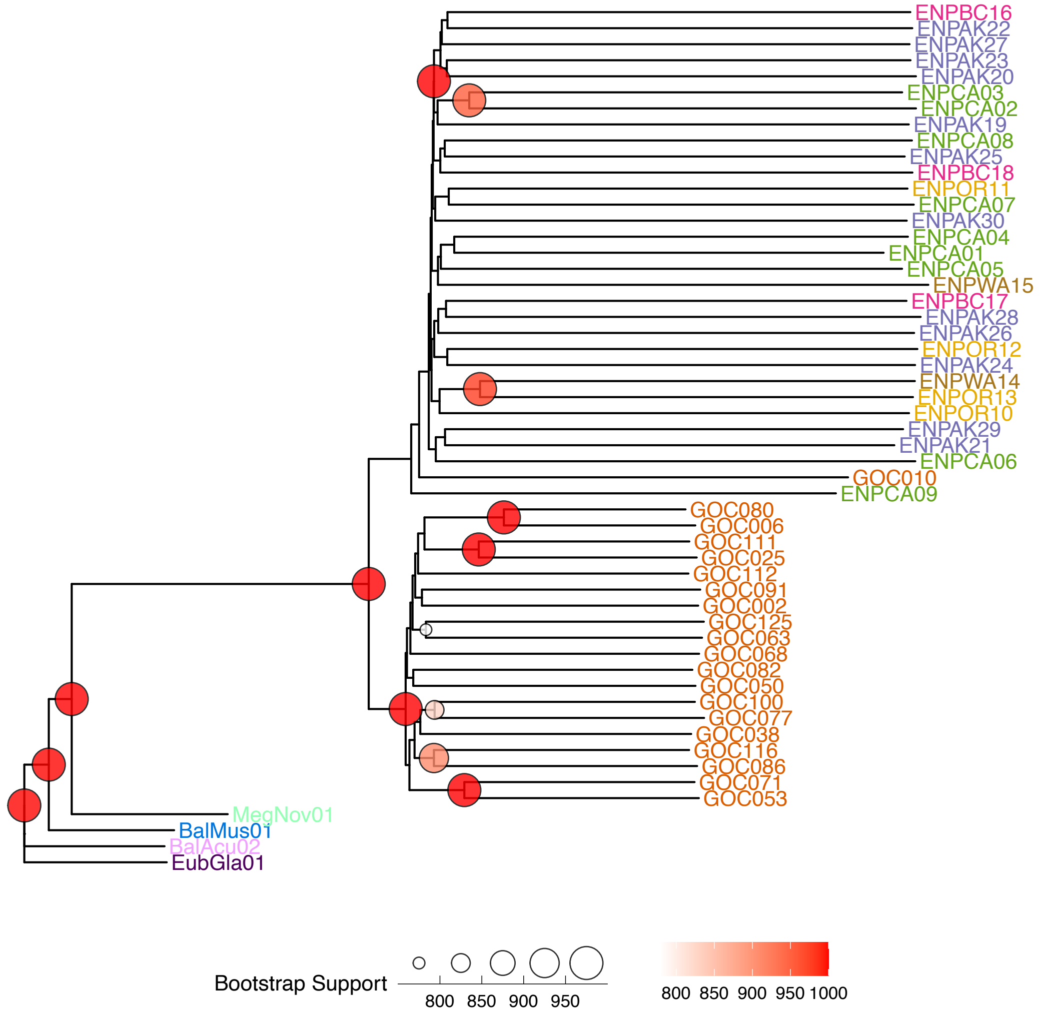

Fig. S5. Neighbor-joining tree. Neighbor-joining tree constructed from a genome-wide pairwise distance matrix containing 50 fin whale and four other baleen whale genomes. GOC individuals form a monophyletic clade, while samples from the different locations within ENP did not. The two admixed samples, ENPCA09 and GOC010, group with the ENP clade but are the first ones to diverge. The tree is rooted at the North Atlantic right whale (EubGla01) and includes other outgroups: minke whale (BalAcu02), blue whale (BalMus01) and humpback whale (MegNov01). The branch length corresponds with genetic distance. 1000 bootstraps were performed. Only nodes with more than 75% support were marked, with larger and darker nodes representing higher support.

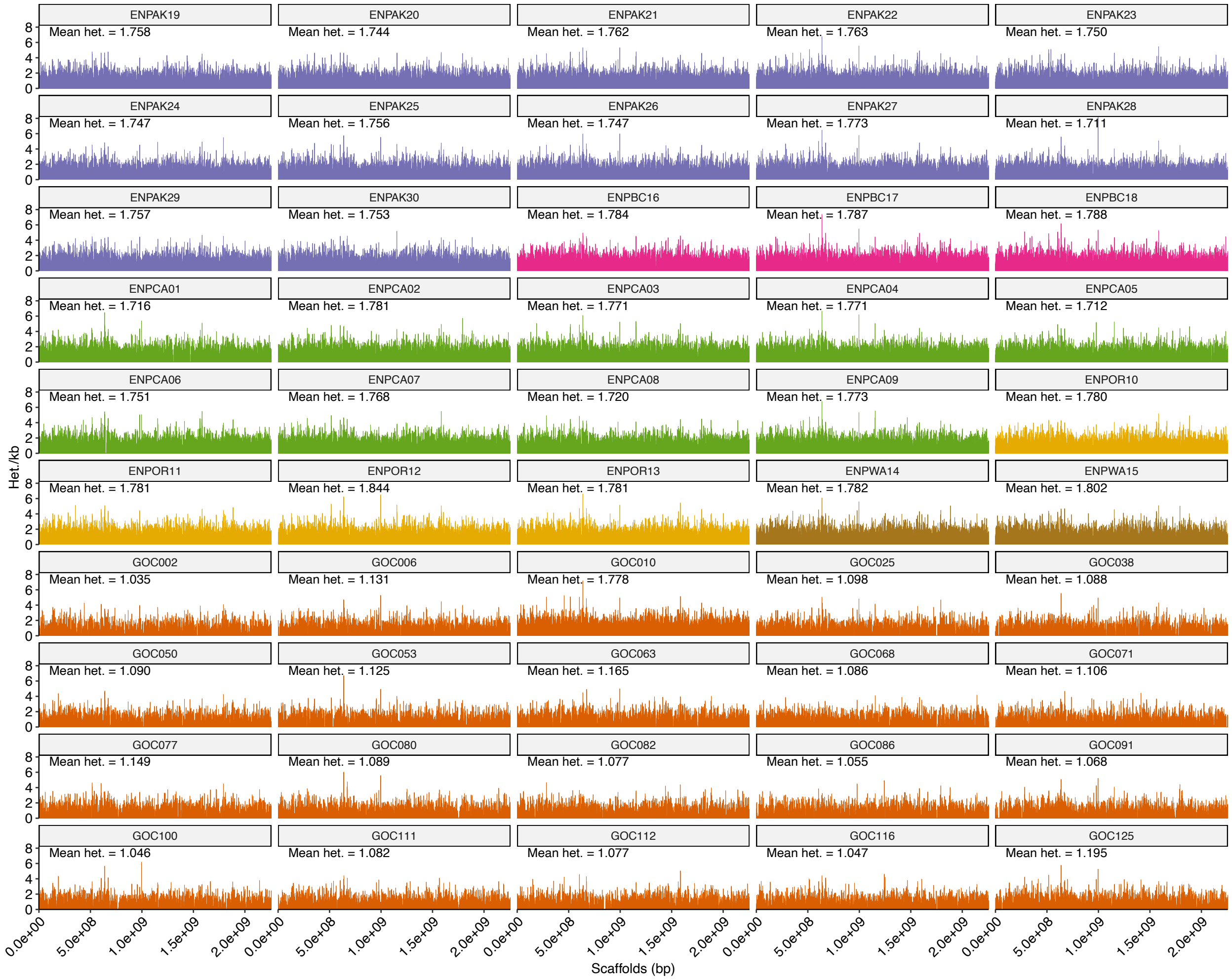

Fig. S6. Genomewide distribution of heterozygosity in all individuals. Genome wide distribution of heterozygosity in all 50 fin whales. The height of the bars stands for per-site heterozygosity in non-overlapping 1 Mb windows across the scaffolds. Colors indicate sampling location (Fig. 1). The mean heterozygosity value is shown for each individual (in units of heterozygotes/kb).

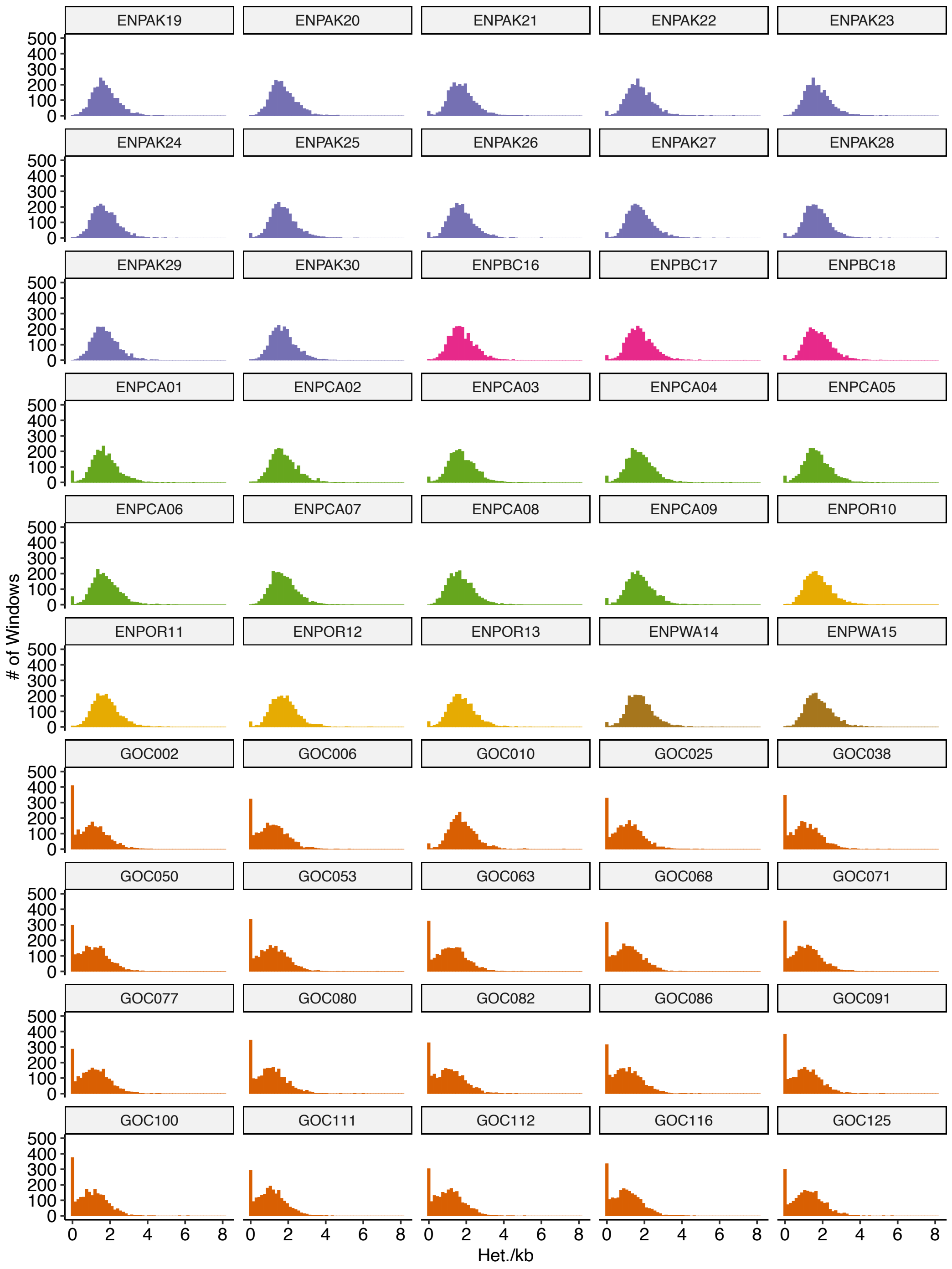

Fig. S7. Histograms of genomewide distribution of heterozygosity in all individuals. Histogram of per-site heterozygosity in non-overlapping 1 Mb windows across the scaffolds in all 50 fin whales. The bin width is set at 0.15 Het/kb. Colors indicate sampling locations (Fig. 1). GOC individuals show enrichment of regions with very low heterozygosity (< 0.075 het/kb with the lowest bin centered at zero).

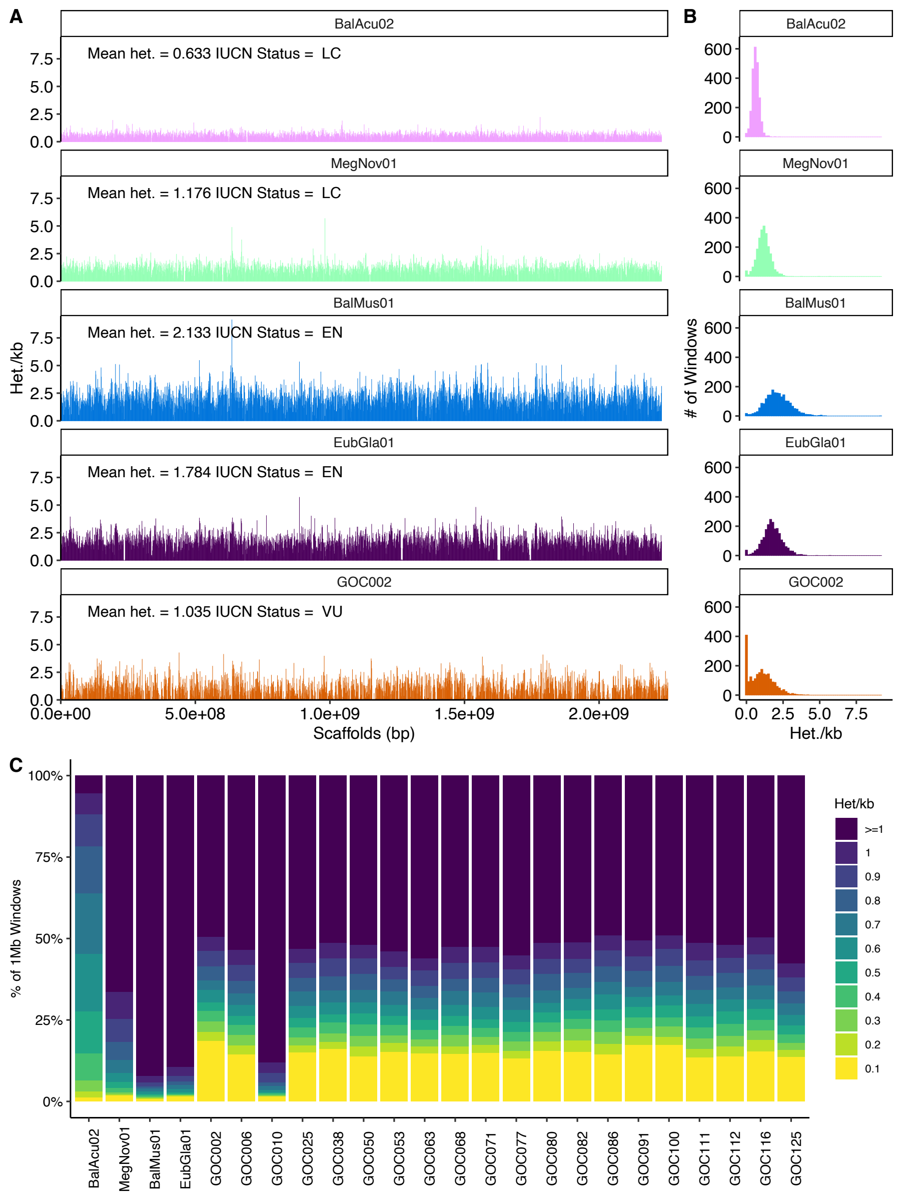

Fig. S8. Comparison of per-site heterozygosity patterns of the Gulf of California fin whales with other baleen whales. **(A)** Per-site heterozygosity in non-overlapping 1-Mb windows across scaffolds shown for a representative individual of the Gulf of California fin whales (GOC002; fin whales are considered vulnerable [VU] by the IUCN), together with individuals from other whale species under different IUCN conservation status: a minke whale (BalAcu02, least concern [LC]), a humpback whale (MegNov01, least concern [LC]), a blue whale (BalMus01, endangered [EN]) and a North Atlantic right whale (EubGla01, endangered [EN]). **(B)** Histogram of the per-window heterozygosity shows that the GOC individual has a higher number of windows with low heterozygosity. **(C)** Stacked barplot showing the distribution of windowed heterozygosity for all GOC fin whales and the other baleen whales. The GOC individuals have a higher proportion of windows with null or very low heterozygosity (0 – 0.1 het/kb) compared with any of the other whale species, representing 13.3% – 18.6% (excluding the admixed GOC010 individual) of the total number of windows for this population.

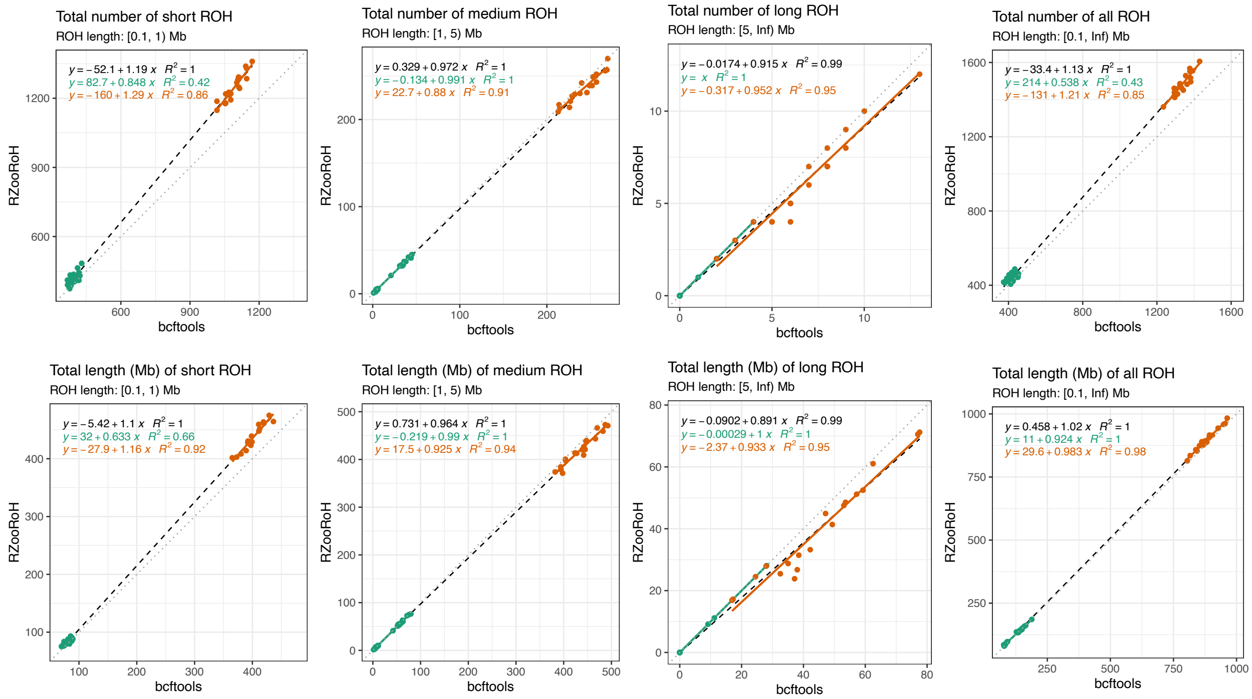

Fig. S9. Comparisons of ROH identified using RZooRoH and bcftools. Comparisons of ROH identified using bcftools and RZooRoH. ROH identified by these methods are highly similar, with RZooRoH identifying more short ROH (ROH < 1Mb) regions and a slightly longer total length for this category. Total number and length (in Mb) of each ROH category identified by the two softwares are plotted. The black dashed line shows the regression for both populations, the green and orange lines show the regression for the ENP and GOC population, respectively, and the gray dotted line shows the diagonal.

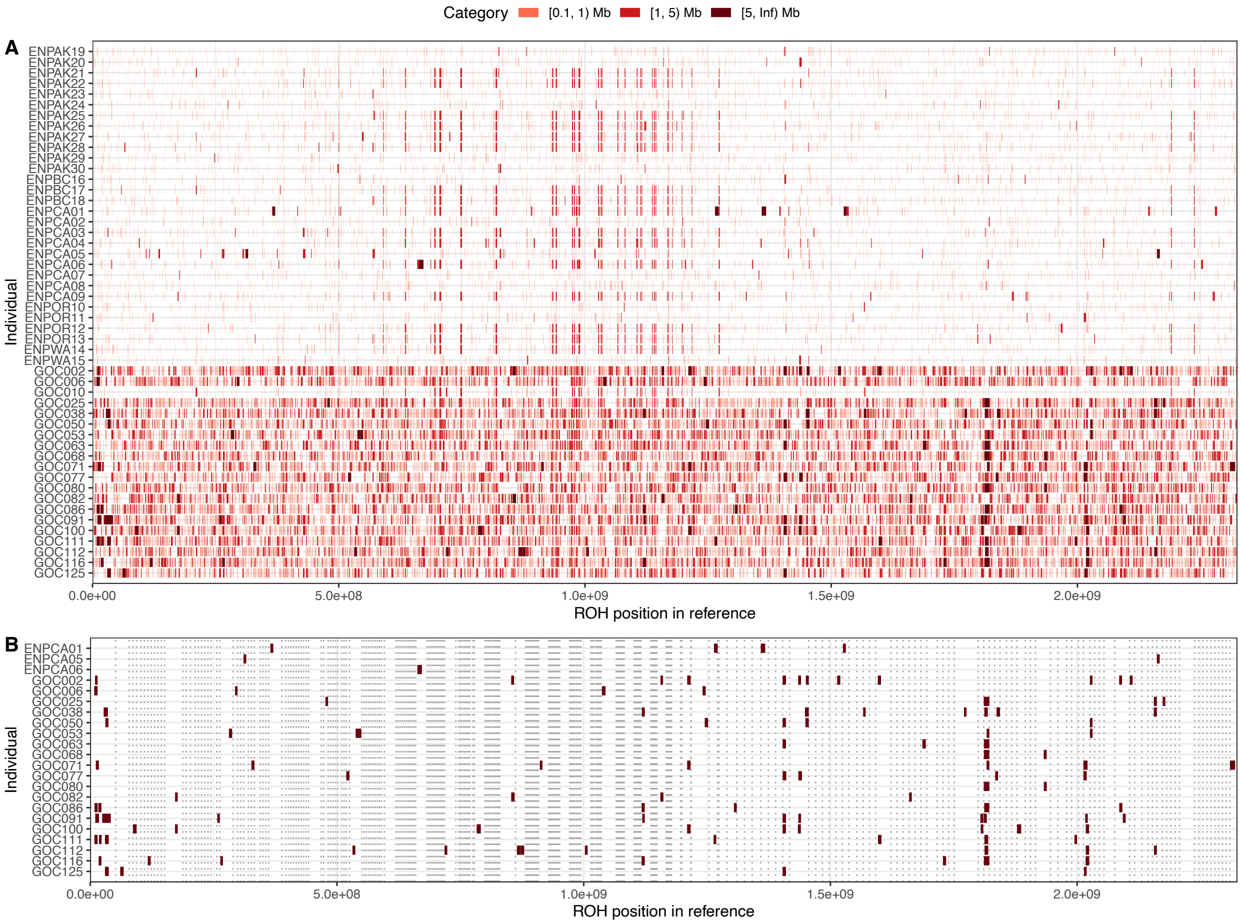

Fig. S10. Genomewide distribution of ROH. Genomewide distribution of the ROH identified using RZooRoH. **(A)** All ROH > 0.1 Mb are visualized across scaffolds, for all fin whale individuals. Darker segments represent longer ROH length categories. **(B)** Only individuals containing ROH > 5 Mb are visualized. The dotted line shows scaffold boundaries.

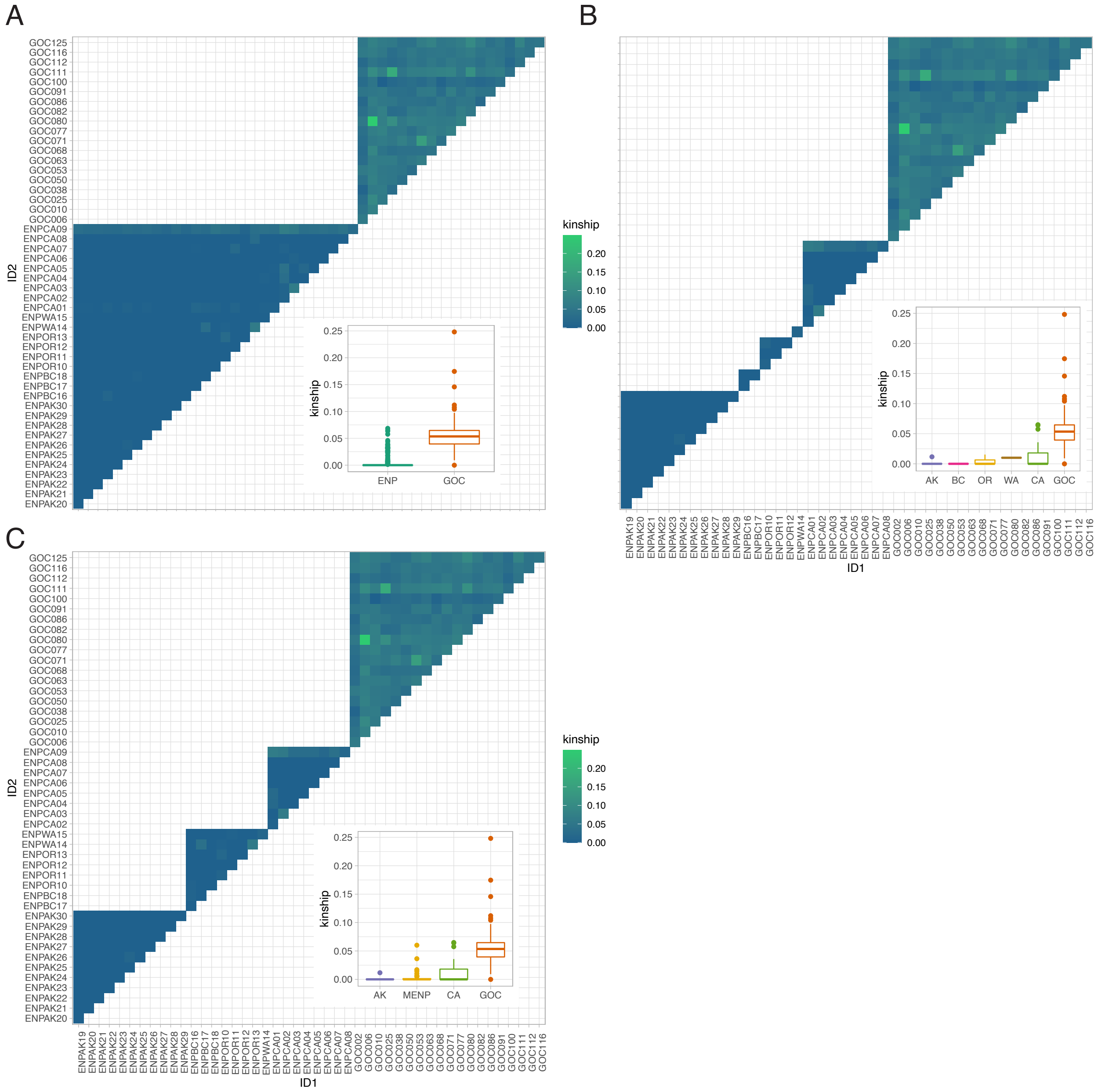

Fig. S11. Kinship matrices within each population or location group. Inset shows the boxplot for pairwise kinship estimates within each group. **(A)** Eastern North Pacific (ENP; N = 30) and Gulf of California (GOC; N=20) populations. **(B)** GOC and sampling groups within the ENP: Alaska (AK; N = 12), British Columbia (BC; N = 3), Oregon (OR; N = 4), Washington (WA; N = 2) and California (CA; N = 9). **(C)** GOC and location groups with similar sample sizes within the ENP: middle ENP includes samples from BC, OR and WA (MENP; N = 9). In the boxplots, the notch indicates the median, and the boxes represent the 25th and 75th percentiles. The whiskers extend to data points no more than 1.5 * IQR (inter-quantile range) from the hinges and the points show outliers beyond the whiskers.

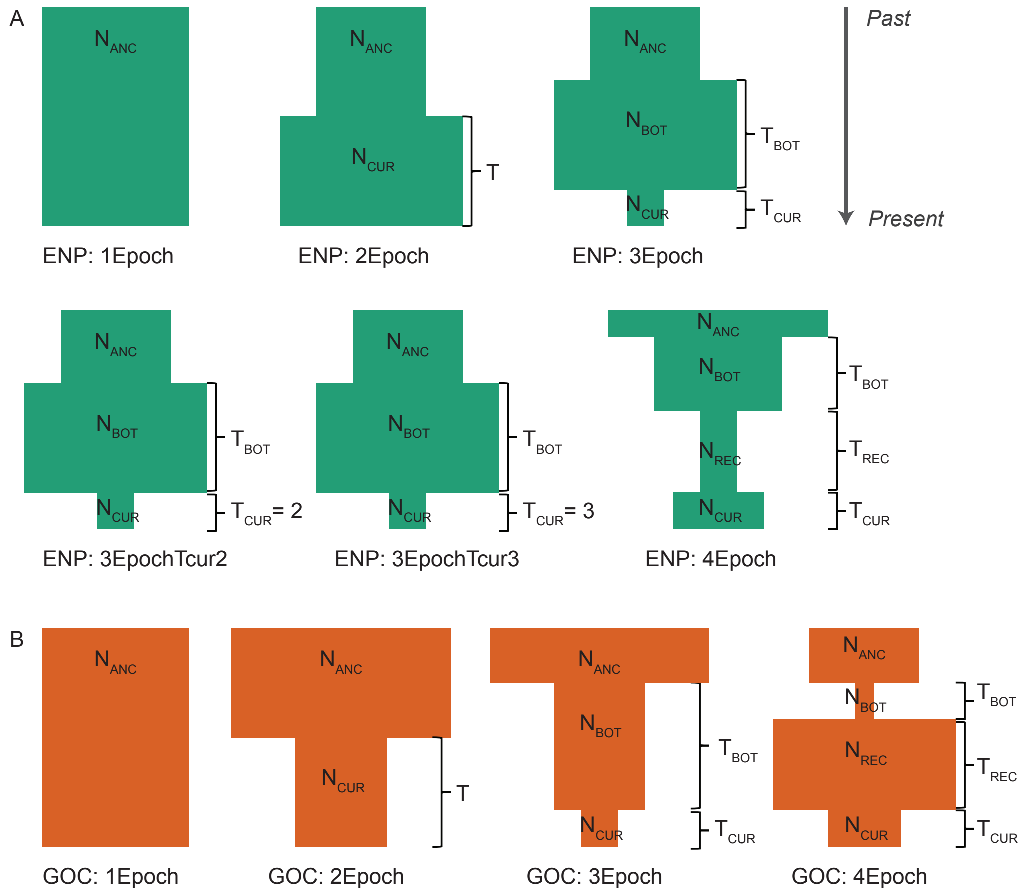

Fig. S12. Single-population demographic model illustrations. Representations of the single-population demographic models used in demographic inference. All models are forward in time. For population size parameters (N_{ANC}, N_{CUR}, etc.), values represent numbers of diploids. For time parameters (T, T_{CUR}, etc.), values represent the number of generations. The sizes or duration of parameters are not to scale but the directions of population size changes (i.e. contraction or expansion) are presented according to the inferred demographic scenarios. Refer to Table S7 for detailed parameter value and uncertainty estimates. **(A)** The demographic models used for the ENP population (from left to right, top to bottom): 1Epoch, single-epoch model with no population size change. 2Epoch, two-epoch model with one size change event (expansion). 3Epoch, three-epoch model with two size change events (expansion then contraction). 3EpochTcur2, three-epoch model with the time since the most recent contraction fixed at two generations. 3EpochTcur3, three-epoch model with the time since the most recent contraction fixed at three generations. 4Epoch, four-epoch model with three size change events (contraction, contraction, expansion). **(B)** The demographic models used for the GOC population (from left to right): 1Epoch, single-epoch model with no population size change. 2Epoch, two-epoch model with one size change event (contraction). 3Epoch, three-epoch model with two size change events (two contractions). 4Epoch, four-epoch model with three size change events (contraction, expansion then contraction).

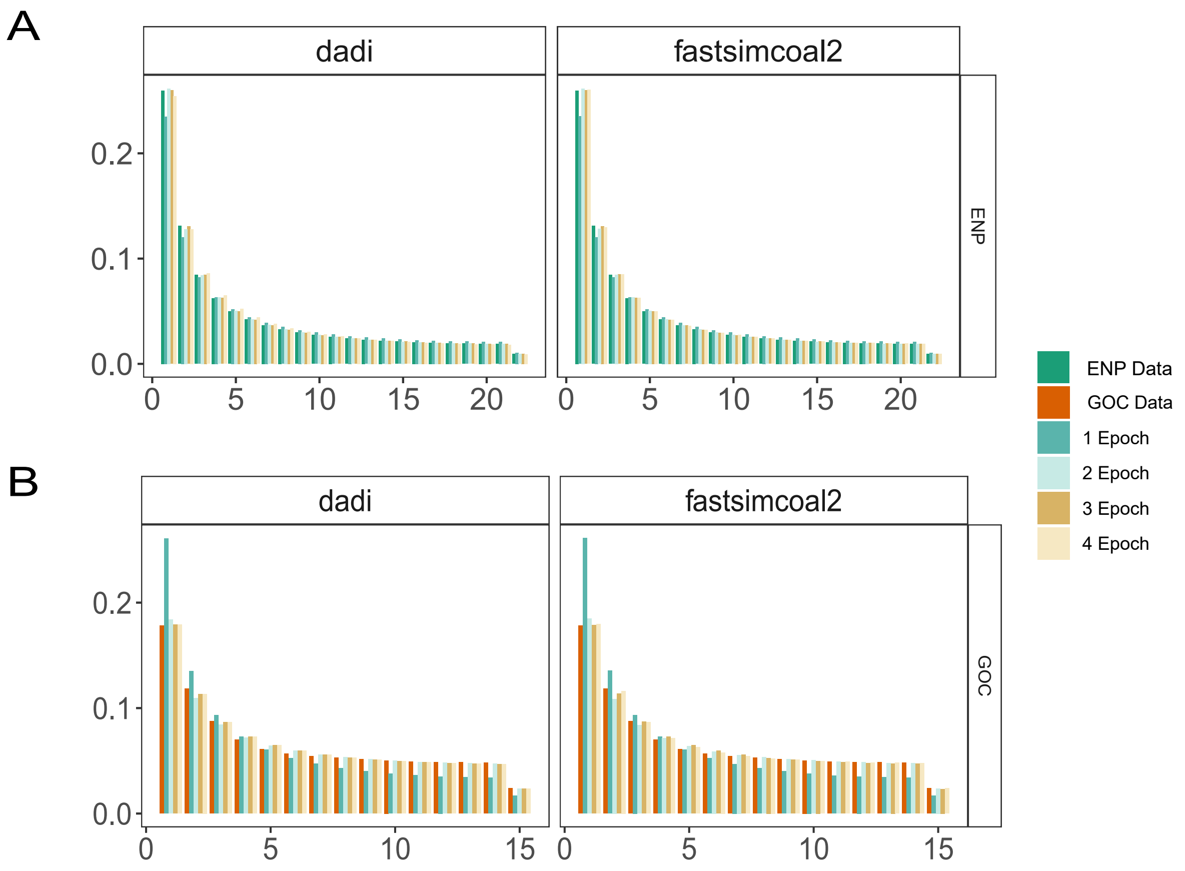

Fig. S13. Fit of the inferred single-population site frequency spectra (SFS) to the observed SFS from the data. Comparison of the inferred SFS for each single-population demographic model (1 to 4 Epoch) obtained with ∂a∂i and fastsimcoal2 with the SFS from the empirical data for **(A)** the Eastern North Pacific population (ENP Data) and **(B)** Gulf of California population (GOC data). Note that for the ENP population the 3 Epoch model fits best, whereas for the GOC population none of the models have a good fit to the data.

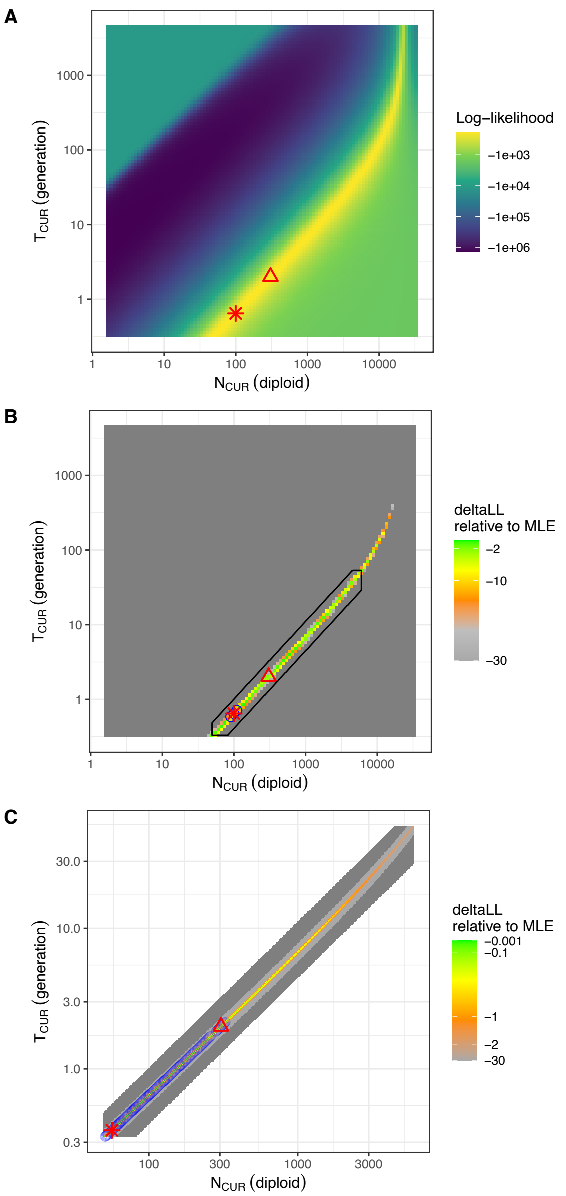

Fig. S14. 3-epoch demographic model grid search for ENP population. Grid search carried out in ∂a∂i for the 3-epoch model in the ENP population. Values for N_{CUR} (current effective population size) and T_{CUR} (time of most recent population size change) are spaced evenly on a log10 scale grid. **(A)** A broad grid search reveals a ridge of likelihoods that are within two units of the maximum likelihood estimate (MLE) from the grid research. **(B)** The same grid research as in **(A)** but plotted under a finer color scale to show in more detail the ridge of high log-likelihoods. **(C)** A zoomed-in grid search conducted within the range of parameter pairs that resulted within two units of the MLE from the initial broad grid search (black box in **B**). We note that there is a consistent yet small (less than 0.1) increase in the log-likelihood favoring a more severe and recent contraction event. In all panels, the red triangle represents the best ∂a∂i estimate from the data (LL = -187.4310), the red asterisk denotes the MLE obtained from the grid search (LL = -187.3507 in **A** and **B**; LL = -187.3377 in **C**) and the blue circles show the parameter pairs that resulted in better log-likelihood compared with the MLE from the data (red triangle).

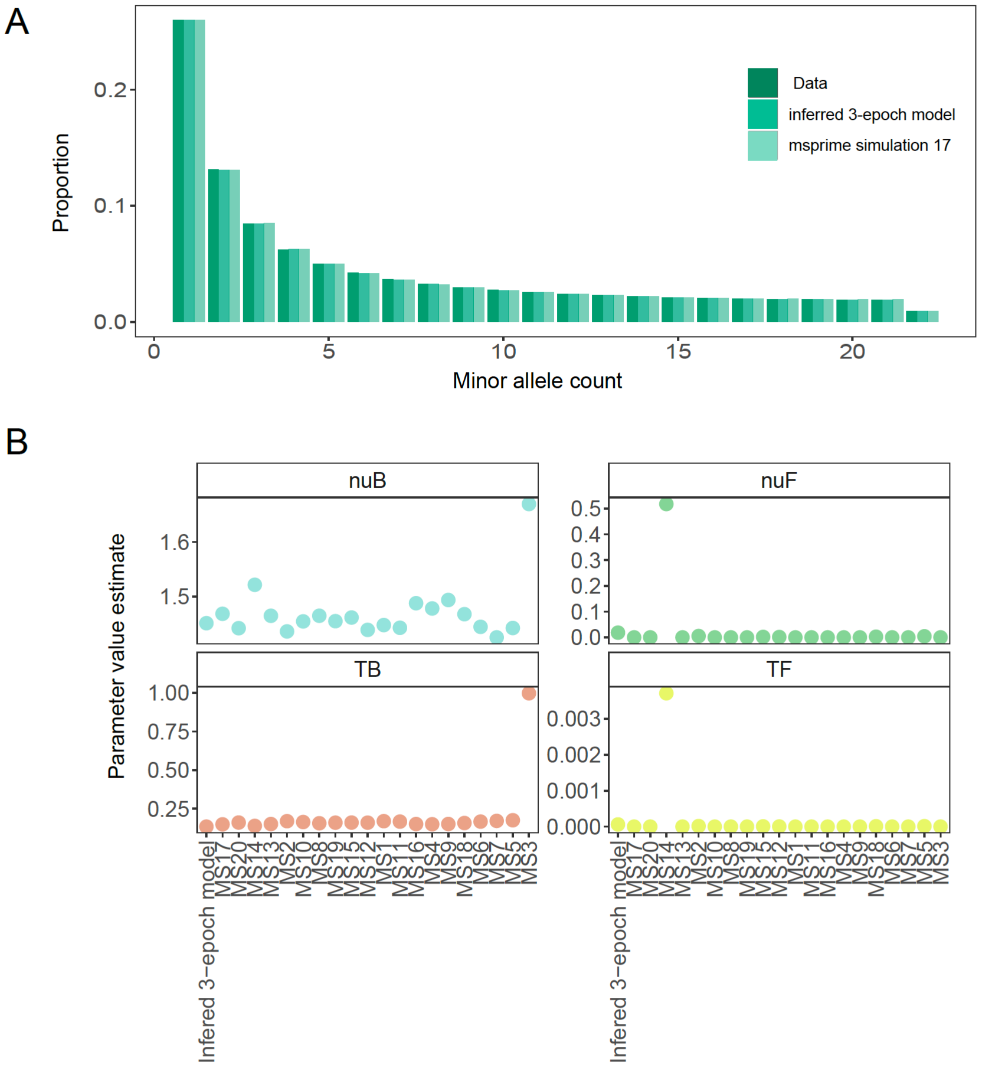

Fig. S15. msprime coalescent SFS simulations of ∂a∂i's 3-epoch model for the ENP population. Demographic inference for 20 msprime coalescent site-frequency spectra (SFS) simulation runs based on our 3-epoch ∂a∂i model for the ENP population. **(A)** Plot showing the fit of the ∂a∂i inferred SFS for the ENP population 3-epoch model (inferred 3-epoch model) and the simulated SFS of a representative coalescent msprime simulation run (msprime simulation 17) to the empirical SFS of the ENP population (Data). **(B)** Plots showing parameter estimations for the inferred ∂a∂i 3-epoch model and for each of the msprime coalescent SFS simulations. *nuB* is the ratio of bottleneck population size (N_{BOT}) to ancient population size (N_{ANC}). *nuF* is the ratio of contemporary (N_{CUR}) to ancient size (N_{ANC}). *TB* is the duration of bottleneck, in units of 2* N_{ANC}, while *TF* denotes the time since bottleneck recovery. We note that despite the variables being named as "bottleneck" sizes, the direction of population size change was not limited. In fact, the model inferred first a population expansion ($nuB > 1$) then a reduction event ($nuF < 1$). The simulated runs are ordered from highest to lowest log-likelihood from left to right. Most of the simulations (except simulation 3, which failed to converge) approximate the magnitude and time of the population reduction (*nuF* and *TF*, respectively), supporting our empirical data results that an extreme reduction occurred recently, most probably due to whaling.

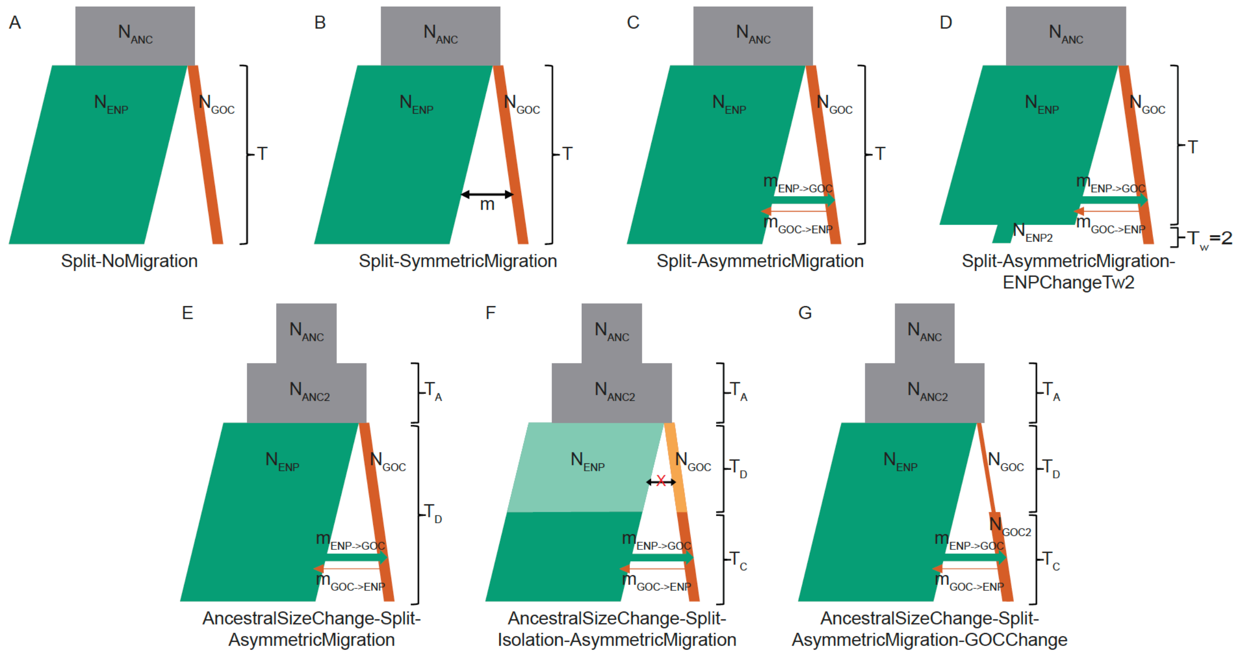

Fig. S16. Two-population demographic model illustrations. Representations of the two-population demographic models used in demographic inference. The sizes or duration of parameters are not to scale, but the directions of population size changes (i.e. contraction or expansion) are presented according to the inferred demographic scenarios. Refer to Table S11 for detailed parameter value and uncertainty estimates. **(A)** A simple population split model with no migration. At time *T*, the ancestral population diverges into the ENP and GOC populations, and the two populations remain isolated. **(B)** An isolation-migration model, the ENP and GOC populations maintain a symmetric migration rate. **(C)** Another isolation-migration model, compared with **(B)**, the populations are allowed to have different values of migration rate (asymmetric migration). **(D)** Based on **(C)**, an ENP population size change event to N_{ENP2} is introduced after population divergence, with a fixed $T_W = 2$ generations before present. This size change event is used to model the impact of whaling bottleneck. **(E)** Based on **(C)**, an ancestral size change event is introduced before population divergence. **(F)** Based on **(E)**, after divergence, an isolation period lasted for T_D, during which there is no migration between the ENP and GOC populations. **(G)** Based on **(E)**, after divergence, the GOC population experiences a size change event happening T_C before current time.

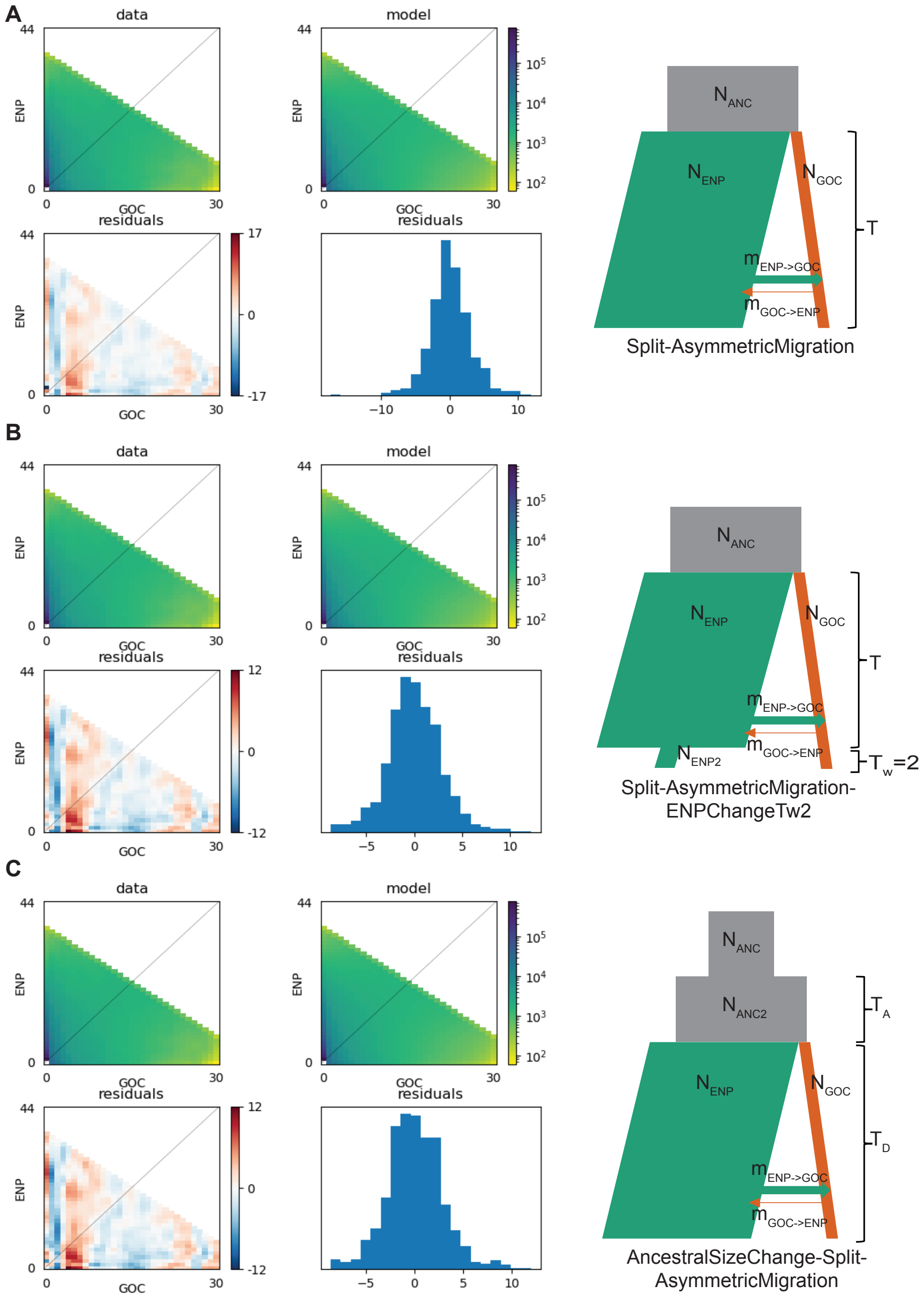

Fig. S17. Two-population demographic model fit to the data. Diagnostic plots for the three best-performing two-population ∂a∂i models. **(A)** Split-AsymmetricMigration; **(B)** Split-AsymmetricMigration-ENPChangeTw2; **(C)** AncestralSizeChange-Split-AsymmetricMigration. Model illustrations are shown in the right panel. For detailed model description, refer to Fig. S16 and Methods. Within each subplot in the left panel, we show the two-population site frequency spectrum (SFS) obtained from the genomic data (top-left); the inferred two-population SFS from the maximum-likelihood iteration of the given model (top-right); the residuals between model and data, with red or blue residuals indicating the model predicts more or fewer alleles, respectively, in a given site-frequency cell (bottom-left) and the histogram of the residuals (bottom-right). Note the improvement of model fit in **(C)** compared with **(A)** or **(B)**.

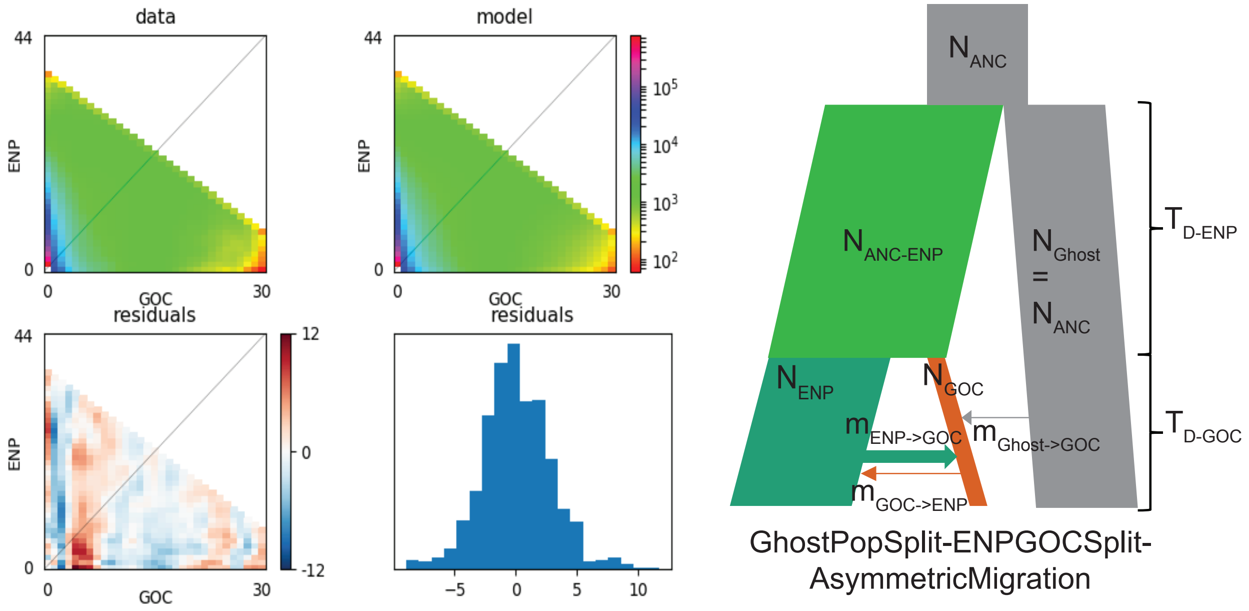

Fig. S18. Ghost-population demographic model fit to the data. (Left) Diagnostic plots for the best-performing ghost-population ∂a∂i model. Within each subplot in the left panel, we show the two-population site frequency spectrum (SFS) obtained from the genomic data (top-left); the inferred two-population SFS from the maximum-likelihood iteration of the given model (top-right); the residuals between model and data, with red or blue residuals indicating the model predicts more or fewer alleles, respectively, in a given site-frequency cell (bottom-left) and the histogram of the residuals (bottom-right). (Right) Model illustrations. For detailed model description refer to Methods.

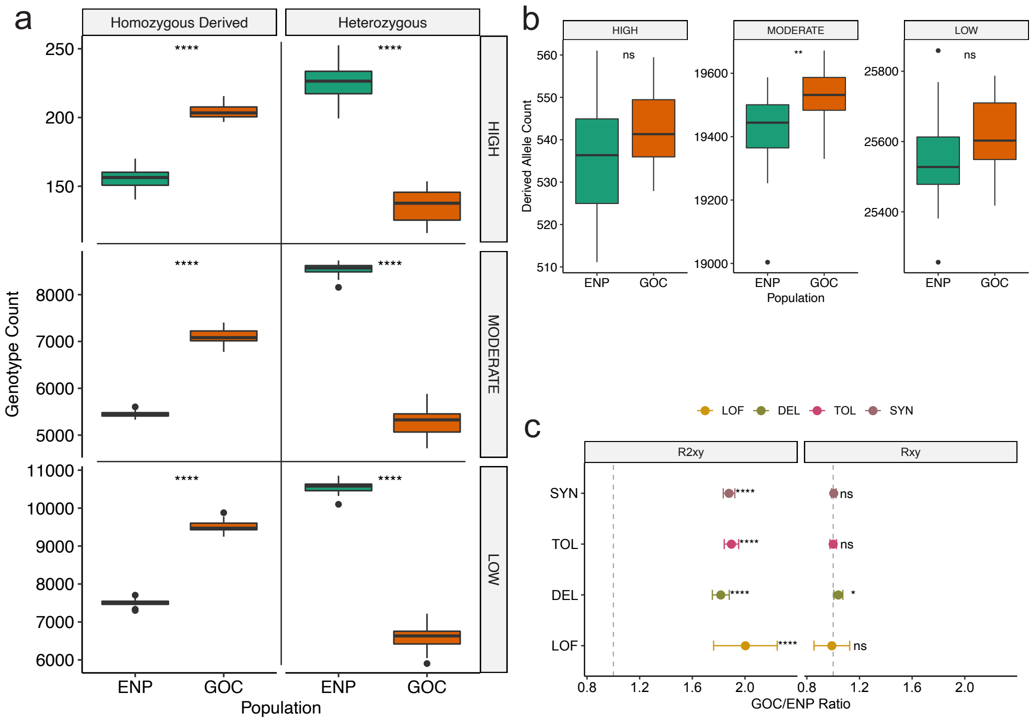

Fig. S19. Deleterious variation categorized using snpEff's impact categories. Deleterious variants were categorized using snpEff's impact classifications of HIGH, MODERATE and LOW. Sample sizes: Gulf of California (GOC) N=17, Eastern North Pacific (ENP) N=27. **(A)** The GOC fin whales contain a similar amount of derived alleles with LOW and HIGH functional impacts and a slight but significantly elevated amount of derived alleles with MODERATE functional impact, compared with the ENP. **(B)** The GOC fin whales contain significantly fewer heterozygous and more homozygous derived genotypes across three impact categories. For (A) and (B) we used two-tailed Mann-Whitney U (MWU) tests without adjustment for multiple testing (MWU test significant values for homozygous derived counts: p_{HIGH} = 2.9e-12, $p_{MODERATE}$ = 2.9e-12, p_{LOW} = 2.9e-12; MWU test significant values for heterozygous counts: p_{HIGH} = 2.9e-12, $p_{MODERATE}$ = 2.9e-12, p_{LOW} = 2.9e-12; MWU test significant values for derived allele count comparisons: p_{HIGH} = 0.1, $p_{MODERATE}$ = 0.0018, p_{LOW} = 0.12 . In the boxplots, the notch indicates the median, and the boxes represent the 25th and 75th percentiles. The whiskers extend to data points no more than 1.5 * IQR (inter-quantile range) from the hinges and the points show outliers beyond the whiskers. **(C)** R_{XY} and R^2_{XY} statistics in the GOC (X) and ENP (Y) populations. $R_{XY} > 1$ (dashed gray line) indicates a relative accumulation of the corresponding mutation category in the GOC population. Similarly, $R^2_{XY} > 1$ indicates relative accumulation of homozygous mutations. The 2x standard error based on the jackknife distribution is denoted as the error bar, the circles in the center of the error bars represent the R_{XY} or R^2_{XY} values. For (C) we used a two-tailed Z-score test without multiple testing adjustment. For (C) we used a two-tailed Z-score test without multiple testing adjustment (Rxy Z-test significant values: p_{HIGH} = 0.66, $p_{MODERATE}$ = 0.31, p_{LOW} = 0.68; R2xy Z-test significant values: p_{HIGH} = 2.81e-23, $p_{MODERATE}$ = 3.73e-255, p_{LOW} = 0). Significance levels: ns, not significant; * $p < 0.05$; ** $p < 0.01$; *** $p < 0.001$; **** $p < 0.0001$.

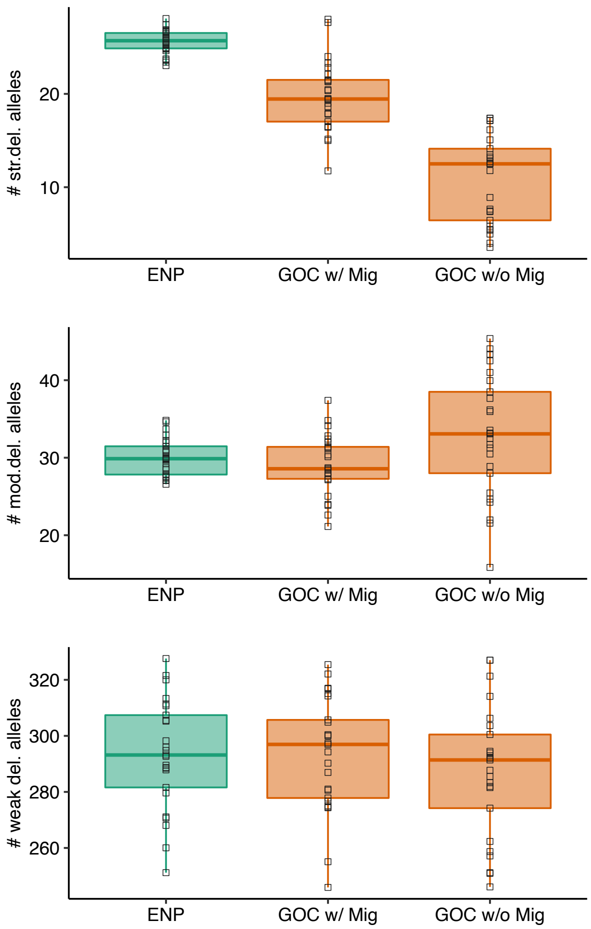

Fig. S20. Slim simulations of deleterious variation. Results for simulations under the two-population model for the ENP and GOC populations, showing the average number of strongly (*s* < -0.01; top panel), moderately (-0.01 < *s* ≤ -0.001; middle panel), and weakly (-0.001< *s* ≤-0.00001; lower panel) deleterious alleles per individual. Each quantity is shown for the ENP and GOC populations at the end of the simulation, including the case where no migration was simulated. For all boxplots, the notch indicates the median, and the boxes represent the 25th and 75th percentiles. The whiskers extend to data points no more than 1.5 * IQR (inter-quantile range) from the hinges and the solid squares show outliers beyond the whiskers. Values from each simulation replicates (n = 25) are overlaid as black squares.

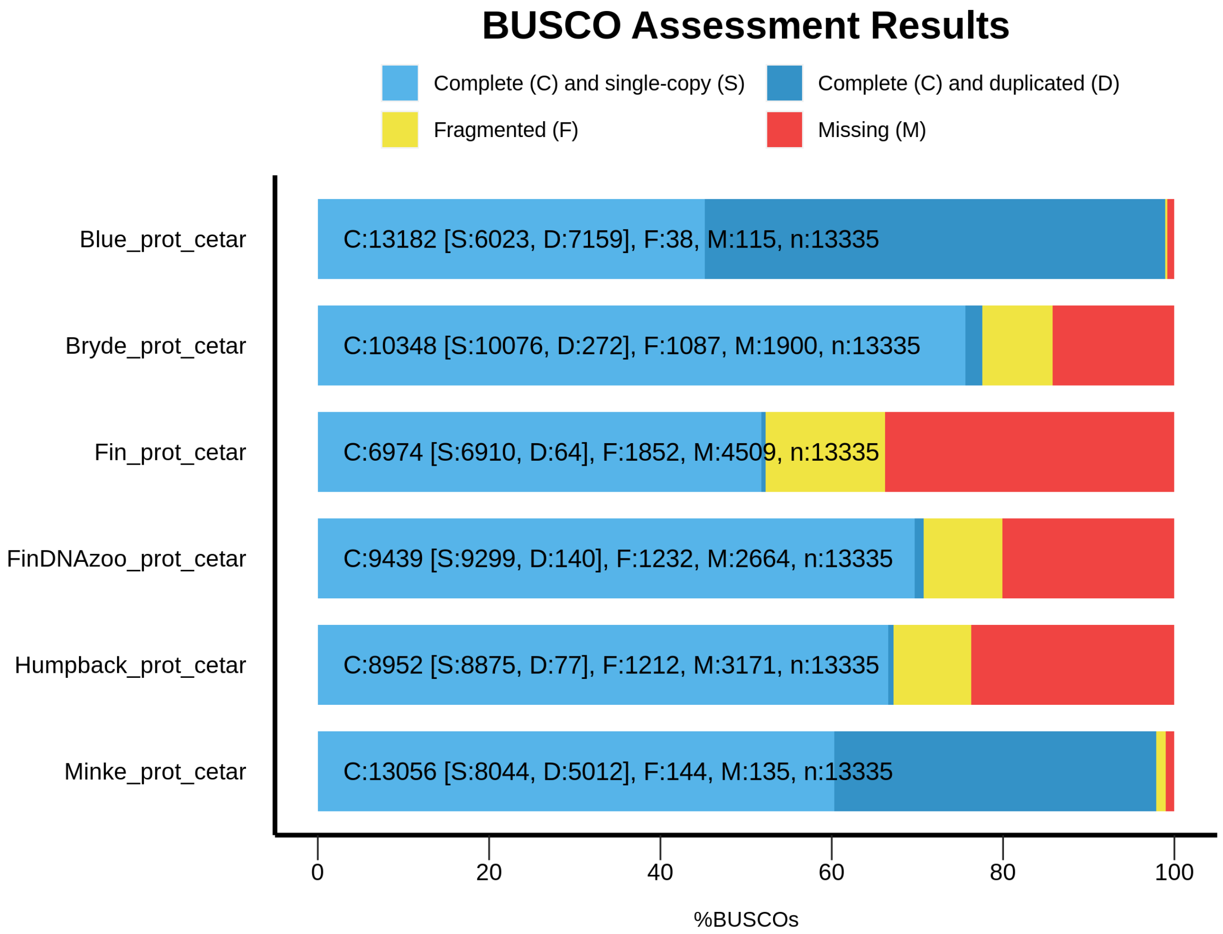

Fig. S21. Comparisons of the BUSCO completeness scores for available protein annotations in the *Balaenopteridae* family. The database used is cetartiodactyla_odb10. The Number of BUSCO genes in this database is 13335. (C)omplete and (S)ingle; (C)omplete and (D)uplicated; (F)ragmented and (M)issing BUSCO proteins are reported. From top to bottom, Blue whale, Rice's whale, Fin whale (NCBI), Fin whale (DNAzoo), Humpback whale and Minke whale assemblies' annotations are compared. For assembly accessions, see Table S16.

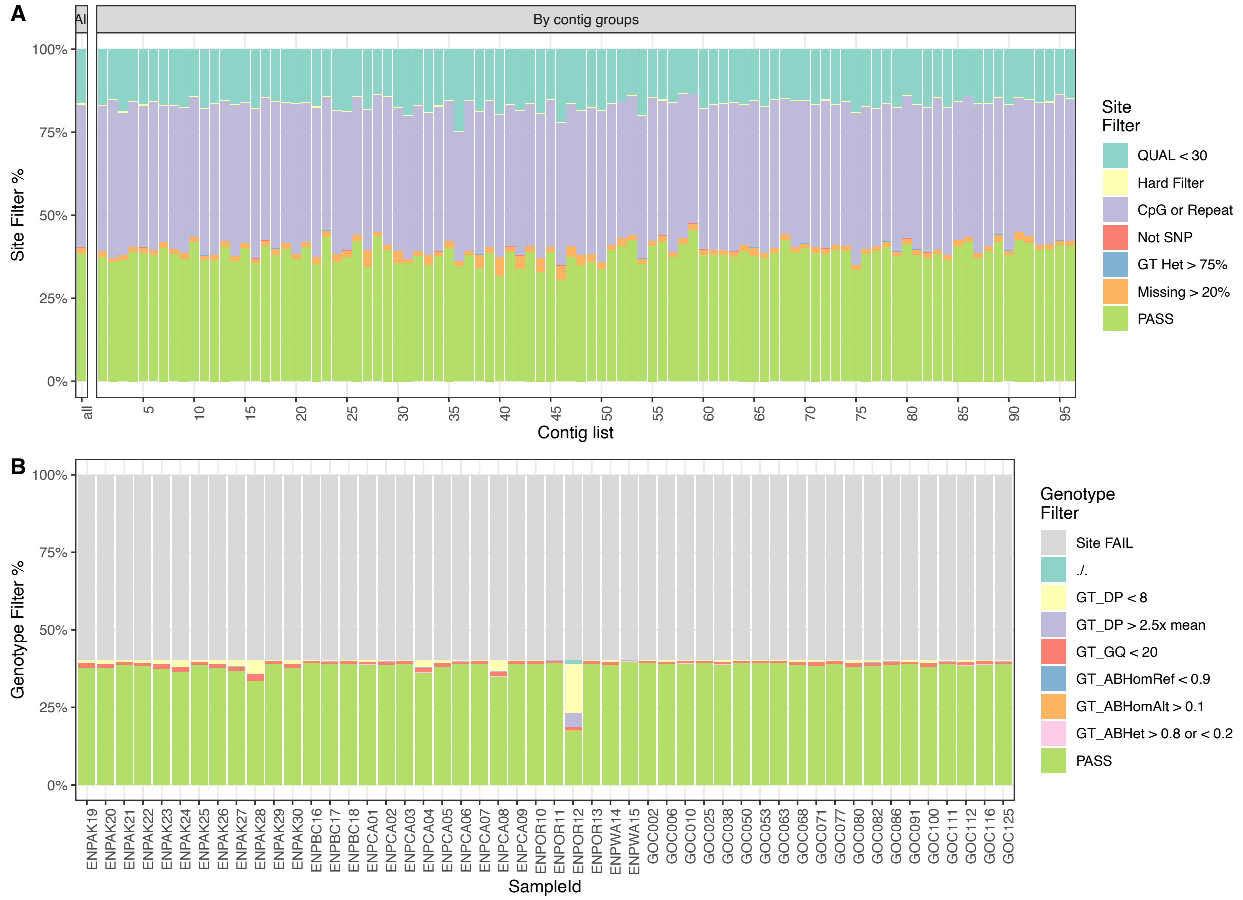

Fig. S22. The proportion of **(A)** sites and **(B)** genotypes that passed (green bar) or failed different filtering criteria. For sites that failed multiple filters, only one filter is counted by the order. For example, if a site has QUAL < 30 (Cyan) and also falls into CpG or repeat region (Purple), it is counted in the QUAL < 30 category. **(A)** QUAL < 30: low Phred score. Hard Filter: failed GATK recommended hard filters (QD < 2.0 || FS > 60.0 || MQ < 40.0 || MQRankSum < -12.5 || ReadPosRankSum < -8.0 || SOR > 3.0). CpG or Repeat: fell within repeat regions identified by WindowMasker[28], RepeatMasker or CpG islands identified by UCSC genome browser (total length: 1,247,900,490 bp). Not SNP: Variant type is not invariant or SNPs, e.g. INDELs. GT Het > 75%: After genotype filter, the number of heterozygous genotypes ("0/1") exceeds 75% of the called genotypes. Missing > 20%; After genotype filter, the number of missing genotypes ("./.") exceeds 20% of the sample size. **(B)** Site FAIL: the site this genotype belongs to failed site-level filters (QUAL < 30, Hard Filter, CpG or Repeat, Not SNP). ./.: this genotype was missing before genotype filtration. GT_DP < 8: genotype depth less than eight. GT_DP > 2.5x mean: genotype depth more than 2.5x mean depth for this sample. GT_GQ < 20: genotype quality less than 20. GT_ABHomRef < 0.9: For a homozygous reference genotype ("0/0") the allelic balance is less than 0.9. GT_ABHomAlt < 0.1: For a homozygous alternate genotype ("1/1") the allelic balance is more than 0.1. GT_ABHet > 0.8 or < 0.2: For a heterozygous genotype ("0/1") the allelic balance is more than 0.8 or less than 0.2. Filters and their criteria are described in detail in the methods.

Supplemental Tables

Table S1. Samples information and sequencing statistics. Summary information of the 50 fin whale samples analyzed and four whole genome sequences from other baleen whale species that were included in our study. The information for each sample includes sample ID, population assignment (Eastern North Pacific [ENP]) and Gulf of California [GOC]), sampling location (Alaska [AK], British Columbia [BC], California [CA], Oregon [OR], Washington [WA], Bahía de la Paz [BLP], Bahía de los Angeles [BLA], Puerto Refugio [PRF], Bahía de Loreto [BLO], Bahía Kino [BK], Bahía de los Frailes [BLF], North of Isla Tiburon [NTB]), year of collection, sex (Females [F], Males [M]), number of reads generated from sequencing, mapping rates to the minke whale reference genome, mean coverage to the minke whale reference, total number of called genotypes, NCBI Sequence Reads Archive accession number, species name and common name.

Sample	Population	Location	Year	Sex	Number Reads	Mapping Rate	Mean Coverage	Number Called Genotypes	SRA Accession Number	Species	Common Name
ENPAK19	ENP	AK	2002	F	4.36E+08	99.07%	25.01	8.54E+08	SRR23615158	*Balaenoptera physalus*	Fin whale
ENPAK20	ENP	AK	2003	F	3.99E+08	99.27%	23.35	8.55E+08	SRR23615157	*Balaenoptera physalus*	Fin whale
ENPAK21	ENP	AK	2003	M	4.97E+08	99.26%	28.89	8.76E+08	SRR23615146	*Balaenoptera physalus*	Fin whale
ENPAK22	ENP	AK	2003	M	4.39E+08	99.29%	25.64	8.67E+08	SRR23615135	*Balaenoptera physalus*	Fin whale
ENPAK23	ENP	AK	2004	F	4.23E+08	99.14%	24.19	8.47E+08	SRR23615123	*Balaenoptera physalus*	Fin whale
ENPAK24	ENP	AK	2004	F	3.75E+08	99.13%	21.79	8.26E+08	SRR23615112	*Balaenoptera physalus*	Fin whale
ENPAK25	ENP	AK	2004	M	4.65E+08	99.29%	27.07	8.74E+08	SRR23615111	*Balaenoptera physalus*	Fin whale
ENPAK26	ENP	AK	2004	M	4.01E+08	99.23%	23.4	8.55E+08	SRR23615110	*Balaenoptera physalus*	Fin whale
ENPAK27	ENP	AK	2004	F	4.57E+08	99.07%	26.63	8.36E+08	SRR23615109	*Balaenoptera physalus*	Fin whale
ENPAK28	ENP	AK	2004	M	3.00E+08	99.04%	17.5	7.69E+08	SRR23615134	*Balaenoptera physalus*	Fin whale
ENPAK29	ENP	AK	2004	F	4.72E+08	99.27%	27.7	8.79E+08	SRR23615156	*Balaenoptera physalus*	Fin whale
ENPAK30	ENP	AK	2004	F	4.24E+08	99.38%	24.65	8.53E+08	SRR23615155	*Balaenoptera physalus*	Fin whale
ENPBC16	ENP	BC	2006	F	4.85E+08	99.09%	28.54	8.83E+08	SRR23615154	*Balaenoptera physalus*	Fin whale
ENPBC17	ENP	BC	2007	M	4.71E+08	99.05%	27.72	8.78E+08	SRR23615153	*Balaenoptera physalus*	Fin whale
ENPBC18	ENP	BC	1998	M	4.79E+08	98.97%	28.2	8.79E+08	SRR23615152	*Balaenoptera physalus*	Fin whale
ENPCA01	ENP	CA	1996	M	5.01E+08	99.33%	29.34	8.75E+08	SRR23615151	*Balaenoptera physalus*	Fin whale

ENPCA02	ENP	CA	2001	F	4.24E+08	99.08%	24.95	8.71E+08	SRR23615150	*Balaenoptera physalus*	Fin whale
ENPCA03	ENP	CA	2001	M	5.12E+08	99.23%	30.05	8.79E+08	SRR23615149	*Balaenoptera physalus*	Fin whale
ENPCA04	ENP	CA	2005	M	3.85E+08	98.99%	22.32	8.25E+08	SRR23615148	*Balaenoptera physalus*	Fin whale
ENPCA05	ENP	CA	2009	F	4.31E+08	99.33%	24.9	8.61E+08	SRR23615147	*Balaenoptera physalus*	Fin whale
ENPCA06	ENP	CA	2014	M	5.16E+08	99.10%	30.14	8.80E+08	SRR23615145	*Balaenoptera physalus*	Fin whale
ENPCA07	ENP	CA	2014	F	4.94E+08	99.26%	28.75	8.79E+08	SRR23615144	*Balaenoptera physalus*	Fin whale
ENPCA08	ENP	CA	2014	F	4.36E+08	99.26%	24.07	7.91E+08	SRR23615143	*Balaenoptera physalus*	Fin whale
ENPCA09	ENP	CA	2017	M	4.86E+08	99.11%	28.68	8.80E+08	SRR23615142	*Balaenoptera physalus*	Fin whale
ENPOR10	ENP	OR	2014	F	5.16E+08	99.16%	29.9	8.79E+08	SRR23615141	*Balaenoptera physalus*	Fin whale
ENPOR11	ENP	OR	2014	F	6.15E+08	98.40%	35.46	8.81E+08	SRR23615140	*Balaenoptera physalus*	Fin whale
ENPOR12	ENP	OR	2005	M	3.71E+08	98.28%	20.23	3.99E+08	SRR23615139	*Balaenoptera physalus*	Fin whale
ENPOR13	ENP	OR	2014	M	5.10E+08	98.94%	29.96	8.80E+08	SRR23615138	*Balaenoptera physalus*	Fin whale
ENPWA14	ENP	WA	2001	M	4.43E+08	99.04%	26.15	8.75E+08	SRR23615137	*Balaenoptera physalus*	Fin whale
ENPWA15	ENP	WA	2001	F	6.71E+08	98.45%	39.2	8.87E+08	SRR23615136	*Balaenoptera physalus*	Fin whale
GOC002	GOC	BLP	2005	N/A	5.61E+08	99.09%	32.95	8.84E+08	SRR23615133	*Balaenoptera physalus*	Fin whale
GOC006	GOC	BLP	2005	N/A	4.95E+08	99.13%	29.19	8.78E+08	SRR23615132	*Balaenoptera physalus*	Fin whale
GOC010	GOC	BLP	2005	N/A	5.42E+08	99.16%	31.92	8.82E+08	SRR23615131	*Balaenoptera physalus*	Fin whale
GOC025	GOC	BLA	2005	N/A	5.25E+08	99.14%	31.06	8.85E+08	SRR23615130	*Balaenoptera physalus*	Fin whale
GOC038	GOC	PRF	2005	N/A	4.30E+08	99.03%	25.55	8.77E+08	SRR23615129	*Balaenoptera physalus*	Fin whale
GOC050	GOC	BLO	2006	N/A	4.66E+08	99.04%	27.47	8.83E+08	SRR23615128	*Balaenoptera physalus*	Fin whale
GOC053	GOC	BLO	2006	N/A	5.20E+08	99.04%	30.75	8.85E+08	SRR23615127	*Balaenoptera physalus*	Fin whale
GOC063	GOC	BLO	2000	N/A	5.21E+08	99.05%	30.68	8.83E+08	SRR23615126	*Balaenoptera physalus*	Fin whale
GOC068	GOC	BLO	2000	N/A	4.43E+08	99.24%	25.99	8.71E+08	SRR23615125	*Balaenoptera physalus*	Fin whale
GOC071	GOC	BLO	2000	N/A	4.24E+08	99.13%	24.84	8.67E+08	SRR23615124	*Balaenoptera physalus*	Fin whale
GOC077	GOC	BLO	2001	N/A	4.23E+08	99.02%	24.97	8.80E+08	SRR23615122	*Balaenoptera physalus*	Fin whale
GOC080	GOC	BLA	1995	N/A	4.49E+08	99.37%	26.43	8.60E+08	SRR23615121	*Balaenoptera physalus*	Fin whale
GOC082	GOC	BLA	2004	N/A	3.94E+08	99.03%	23.18	8.66E+08	SRR23615120	*Balaenoptera physalus*	Fin whale
GOC086	GOC	BLA	2004	N/A	4.03E+08	99.13%	23.76	8.74E+08	SRR23615119	*Balaenoptera physalus*	Fin whale
GOC091	GOC	BLA	2004	N/A	4.24E+08	98.98%	25.14	8.79E+08	SRR23615118	*Balaenoptera physalus*	Fin whale

GOC100	GOC	BK	1999	N/A	3.63E+08	99.08%	21.55	8.65E+08	SRR23615117	*Balaenoptera physalus*	Fin whale
GOC111	GOC	BK	2005	N/A	5.19E+08	99.28%	30.35	8.77E+08	SRR23615116	*Balaenoptera physalus*	Fin whale
GOC112	GOC	BK	2005	N/A	4.85E+08	99.09%	28.48	8.69E+08	SRR23615115	*Balaenoptera physalus*	Fin whale
GOC116	GOC	BLF	2003	N/A	4.13E+08	99.06%	24.37	8.77E+08	SRR23615114	*Balaenoptera physalus*	Fin whale
GOC125	GOC	NTB	2003	N/A	4.70E+08	99.02%	27.71	8.80E+08	SRR23615113	*Balaenoptera physalus*	Fin whale
EubGla01	N/A	N/A	N/A	N/A	3.72E+08	94.94%	12.64	6.20E+08	SRR5665640	*Eubalaena glacialis*	North Atlantic right whale
BalAcu02	N/A	N/A	N/A	N/A	4.70E+08	98.86%	18.28	6.12E+08	SRR1802584	*Balaenoptera acutorostrata*	Minke whale
BalMus01	N/A	N/A	N/A	N/A	1.12E+09	98.61%	44.36	8.68E+08	SRR5665644	*Balaenoptera musculus*	Blue whale
MegNov01	N/A	N/A	N/A	N/A	5.40E+08	97.36%	25.77	8.28E+08	SRR5665639	*Megaptera novaeangliae*	Humpback whale

Table S2. Comparison of parameters and statistics derived from the genotyping pipeline using the minke whale and fin whale genomes as the reference. Total sequence length: the sequence length of the genome; Total scaffold length used for genotyping: the sequence length after eliminating the sequences in scaffolds less than 1 Mb long; Exclude percentage of the genome: the percentage of the genome discarded because it was in scaffolds less than 1 Mb long; Average mapping rate: the average proportion of reads mapped to the reference genome; Average number of heterozygotes sites: the mean number of heterozygote sites identified during genotyping; Average number of called sites: the average number of sites called by the genotyping pipeline.

Parameter	Reference Genome	
	Minke whale	Fin whale
Total sequence length	2,431,687,698 bp	2,410,646,746 bp
Total scaffold length used for genotyping	2,324,429,847 bp	2,319,356,520 bp
Excluded percentage of genome	4.41%	3.78%
Average mapping rate	99.09%	99.49%
Average number of heterozygous sites	1,290,413.36	1,457,881.4
Average number of called sites	857,879,621.82	1,046,290,425.1

Table S3. Pairwise F_{ST} values. Pairwise F_{ST} estimated from genome-wide biallelic sites (30,350 SNPs) pruned for linkage disequilibrium and minor allele frequency > 0.1. Three levels of subpopulation partitions were tested: 1) population (ENP – GOC $p = 0.001$), 2) merged locations in Middle Eastern North Pacific (MENP) (AK – MEP $p = 0.004$; AK – CA $p = 0.002$; AK – GOC $p = 0.001$; MENP – CA $p = 0.010$; MENP – GOC $p = 0.001$ and CA – GOC $p = 0.001$) and 3) all locations. The MENP category combined samples from BC, WA and OR. The significance of F_{ST} was estimated using 999 permutations without adjustment for multiple comparisons. Significance levels: * $p < 0.05$, ** $0.005 > p > 0.001$, *** $p = 0.001$. Absence of an asterisk after F_{ST} values means no significance estimate was performed due to a low sample size for those comparisons. Due to the low sample size in BC (N = 3), WA (N = 4) and OR (N = 2), there were insufficient sample origin combinations for permutation tests in all locations and the significance was not estimated.

**By Population**					
	GOC				
ENP	0.073***				
**By Merged Locations**					
	MENP	CA	GOC		
AK	0.0038**	0.0046**	0.0906***		
MENP	NA	0.0053*	0.0980***		
CA	NA	NA	0.0807***		
**By Locations**					
	BC	WA	OR	CA	GOC
AK	0.0024	0.0044	0.0057	0.0046	0.0906
BC	NA	-0.0003	0.0076	0.0069	0.1265
WA	NA	NA	-0.0040	0.0081	0.1465
OR	NA	NA	NA	0.0071	0.1249
CA	NA	NA	NA	NA	0.0807

Table S4. F_{ROH} values per individual. The proportion of the genome containing different length categories of ROH (F_{ROH}) for each individual. Minimum ROH segments length cutoffs are set at 100 kb, 1 Mb and 5 Mb. The RZooRoH's and bcftools' output are very similar across individuals and length cutoffs.

	ZooRoH			Bcftools		
**Sample**	$F_{ROH > 100kb}$	$F_{ROH > 1Mb}$	$F_{ROH > 5Mb}$	$F_{ROH > 100kb}$	$F_{ROH > 1Mb}$	$F_{ROH > 5Mb}$
**ENPAK19**	0.0356	0.0030	NA	0.0340	0.0025	NA
**ENPAK20**	0.0359	0.0035	NA	0.0336	0.0035	NA
**ENPAK21**	0.0574	0.0230	NA	0.0580	0.0241	NA
**ENPAK22**	0.0604	0.0233	NA	0.0598	0.0227	NA
**ENPAK23**	0.0356	0.0013	NA	0.0350	0.0019	NA
**ENPAK24**	0.0376	0.0030	NA	0.0351	0.0025	NA
**ENPAK25**	0.0610	0.0230	NA	0.0610	0.0230	NA
**ENPAK26**	0.0619	0.0241	NA	0.0624	0.0240	NA
**ENPAK27**	0.0613	0.0250	NA	0.0614	0.0261	NA
**ENPAK28**	0.0651	0.0261	NA	0.0650	0.0271	NA
**ENPAK29**	0.0365	0.0007	NA	0.0331	0.0007	NA
**ENPAK30**	0.0382	0.0033	NA	0.0362	0.0039	NA
**ENPBC16**	0.0369	0.0044	NA	0.0345	0.0044	NA
**ENPBC17**	0.0574	0.0219	NA	0.0569	0.0224	NA
**ENPBC18**	0.0588	0.0236	NA	0.0582	0.0236	NA
**ENPCA01**	0.0798	0.0449	0.0121	0.0810	0.0464	0.0121
**ENPCA02**	0.0380	0.0024	NA	0.0363	0.0024	NA
**ENPCA03**	0.0631	0.0272	NA	0.0631	0.0267	NA
**ENPCA04**	0.0681	0.0322	NA	0.0682	0.0325	NA
**ENPCA05**	0.0586	0.0226	0.0048	0.0548	0.0230	0.0048
**ENPCA06**	0.0695	0.0352	0.0039	0.0707	0.0347	0.0039
**ENPCA07**	0.0366	0.0012	NA	0.0349	0.0012	NA
**ENPCA08**	0.0416	0.0015	NA	0.0388	0.0020	NA
**ENPCA09**	0.0663	0.0293	NA	NA	NA	NA
**ENPOR10**	0.0344	0.0013	NA	0.0339	0.0017	NA
**ENPOR11**	0.0420	0.0042	NA	0.0397	0.0049	NA
**ENPOR12**	0.0671	0.0262	NA	NA	NA	NA
**ENPOR13**	0.0627	0.0262	NA	0.0644	0.0267	NA
**ENPWA14**	0.0592	0.0222	NA	0.0595	0.0222	NA
**ENPWA15**	0.0371	0.0037	NA	0.0348	0.0039	NA

**GOC002**	0.4230	0.2341	0.0306	0.4144	0.2429	0.0334
**GOC006**	0.3774	0.1993	0.0110	0.3736	0.2062	0.0140
**GOC010**	0.0541	0.0251	NA	NA	NA	NA
**GOC025**	0.3824	0.1881	0.0120	0.3796	0.2021	0.0120
**GOC038**	0.3938	0.2117	0.0226	0.3890	0.2180	0.0255
**GOC050**	0.3846	0.1848	0.0135	0.3781	0.1903	0.0166
**GOC053**	0.3806	0.1960	0.0124	0.3771	0.2053	0.0151
**GOC063**	0.3670	0.1914	0.0105	0.3630	0.1993	0.0105
**GOC068**	0.3692	0.1849	0.0073	0.3608	0.1911	0.0073
**GOC071**	0.3761	0.1932	0.0209	0.3671	0.1967	0.0231
**GOC077**	0.3592	0.1752	0.0143	0.3512	0.1825	0.0182
**GOC080**	0.3902	0.2050	0.0074	0.3837	0.2149	0.0074
**GOC082**	0.3836	0.2108	0.0103	0.3754	0.2183	0.0160
**GOC086**	0.3941	0.1958	0.0178	0.3841	0.2034	0.0212
**GOC091**	0.4137	0.2211	0.0303	0.4108	0.2338	0.0331
**GOC100**	0.4124	0.2288	0.0263	0.4081	0.2387	0.0269
**GOC111**	0.3785	0.1790	0.0193	0.3717	0.1914	0.0203
**GOC112**	0.3827	0.1851	0.0220	0.3710	0.1942	0.0246
**GOC116**	0.4059	0.2016	0.0204	0.3988	0.2142	0.0228
**GOC125**	0.3502	0.1769	0.0115	0.3462	0.1858	0.0164

Table S5. Summary of demographic models' performances. *K:* the number of estimated parameters used in the corresponding model. Data Log-likelihood: the best possible log-likelihood of the empirical site-frequency spectrum (SFS) fit to itself. Log-likelihood: the best log-likelihood obtained for each particular model, measuring the fit of the model to the empirical data, and thus the performance of the model. AIC: the Akaike information criterion is calculated using the equation AIC = 2*K - 2*Log-likelihood. Convergence of parameters is also reported. Selected models are indicated in the last column. See Methods for full descriptions of the models and Figs. S12, S16 for model illustrations.

		∂a∂i						fastsimcoal2					
**Population**	**Model**	**K**	**Data Log-likelihood**	**Log-likelihood**	**AIC**	**Converged**	**Selected**	**K**	**Data Log-likelihood**	**Log-likelihood**	**AIC**	**Converged**	**Selected**
ENP	1Epoch	0	-147.84	-11193.13	22386.26	Yes		1	-12448596.68	-12453208.82	24906419.64	No	
ENP	2Epoch	2	-147.84	-336.30	676.59	Yes		3	-12448596.68	-12448667.78	24897341.57	Yes	
ENP	3Epoch	4	-147.84	-187.43	382.86	Yes	Yes	5	-12448596.68	-12448610.07	24897230.14	Yes	Yes
ENP	3EpochTcur2	3	-147.84	-187.39	380.78	Yes		4	-12448596.68	-12448608.98	24897225.97	Yes	
ENP	3EpochTcur3	3	-147.84	-187.44	380.89	Yes		4	-12448596.68	-12448606.37	24897220.74	Yes	
ENP	4Epoch	6	-147.84	-1490.68	2993.37	Yes		7	-12448596.68	-12448618.67	24897251.34	Yes	
GOC	1Epoch	0	-99.48	-49253.16	98506.32	Yes		1	-6076602.17	-6098012.27	12196026.53	No	
GOC	2Epoch	2	-99.48	-1251.26	2506.52	Yes		3	-6076602.17	-6077059.96	12154125.93	Yes	
GOC	3Epoch	4	-99.48	-776.67	1561.34	No		5	-6076602.17	-6076862.27	12153734.54	No	
GOC	4Epoch	6	-99.48	-149.71	311.42	No		7	-6076602.17	-6076659.27	12153332.53	No	
ENP-GOC	Split-NoMigration	3	-3314.79	-115372.25	230750.51	No		4	-15378117.61	-15427737.64	30855483.28	No	
ENP-GOC	Split-SymmetricMigration	4	-3314.79	-63126.52	126261.03	Yes		5	-15378117.61	-15403887.53	30807785.06	No	
ENP-GOC	Split-AsymmetricMigration	5	-3314.79	-6419.80	12849.60	Yes		6	-15378117.61	-15379488.52	30758989.04	No	
ENP-GOC	Split-AsymmetricMigration-ENPChangeTw2	6	-3314.79	-6258.03	12528.06	Yes		7	-15378117.61	-15379411.77	30758837.54	No	
ENP-GOC	AncestralSizeChange-Split-AsymmetricMigration	7	-3314.79	-6243.37	12500.73	Yes	Yes	8	-15378117.61	-15379405.93	30758827.86	Yes	Yes
ENP-GOC	AncestralSizeChange-Split-Isolation-AsymmetricMigration	8	-3314.79	-5236.99	10489.97	No		9	-15378117.61	-15379178.35	30758374.7	No	
ENP-GOC	AncestralSizeChange-Split-AsymmetricMigration-GOCChange	9	-3314.79	-5137.64	10293.28	No		10	-15378117.61	-15379402.62	30758825.25	No	

Table S6. Likelihood ratio test (LRT) between nested single-population models. LRT results for the comparison between nested ∂a∂i models are shown below the diagonal and the results for fastsimcoal2 nested models are shown above the diagonal. **(A)** For the Eastern North Pacific population, the LRT results show that, for both inference methods, the 3-epoch model, which represents the whaling model, is significantly better than the 2-epoch model, while a 4-epoch model is not better than the 3-epoch model. The LRT significance was evaluated with a two-sided chi-square test (χ^2) without multiple comparison adjustment with two degrees of freedom, which was the number of parameter differences between models. (χ^2 test for ENP ∂a∂i models: $p_{1vs2epochs} = 0$, $p_{2vs3epochs} = 2.22e\text{-}65$, $p_{3vs4epochs} = 1$. χ^2 test for ENP fastsimcoal2 models: $p_{1vs2epochs} = 0$, $p_{2vs3epochs} = 8.64e\text{-}26$, $p_{3vs4epochs} = 1$). **(B)** For the Gulf of California population, the LRT results indicate that both inference methods find that the 3-epoch and 4-epoch models have significantly better likelihoods than the 2-epoch model. However, the parameter estimations for both of these models are not consistent between ∂a∂i and fastsimcoal2, and lack convergence (See Tables S5 and S7), which could indicate overparameterization of these models (χ^2 test for GOC ∂a∂i models: $p_{1vs2epochs} = 0$, $p_{2vs3epochs} = 1.07e\text{-}206$, $p_{3vs4epochs} = 3.71e\text{-}273$. χ^2 test for GOC fastsimcoal2 models: $p_{1vs2epochs} = 0$, $p_{2vs3epochs} = 1.39e\text{-}86$, $p_{3vs4epochs} = 6.89e\text{-}89$. Significance levels: * $p < 1.0e\text{-}25$, ** $p < 1.0e\text{-}50$, *** $p < 1.0e\text{-}100$, the absence of asterisks identifies $p = 1$.

A)

	1Epoch	2Epoch	3Epoch	4Epoch
1Epoch	–	9082.08***		
2Epoch	21713.67***	–	115.42*	
3Epoch		297.7325**	–	-17.2
4Epoch			-2606.505	–

B)

	1Epoch	2Epoch	3Epoch	4Epoch
1Epoch	–	41904.62***		
2Epoch	96003.79***	–	395.38**	
3Epoch		948.5171***	–	406**
4Epoch			1254.587***	–

Table S7. Demographic parameter estimates for the single-population models. The estimates are based on the analysis of the single-population SFS from the Eastern North Pacific (ENP) or Gulf of California (GOC) populations. The demographic models are identified in the blue header lines. If a model contains fixed parameters, it is denoted as "value (Fixed)" in the estimate column. The parameter estimates and 95% Confidence Intervals derived from ∂a∂i or fastsimcoal2 are reported side by side. For population size parameters (N_{ANC}, N_{BOT}, etc.), all values are in units of number of diploids. For time parameters (T, T_{CUR}, etc.), all values are in units of generations. For time parameters derived from fastsimcoal2, values are additionally converted to time intervals forward in time for better comparisons with ∂a∂i output (e.g. in three-epoch models, T_{BOT} = TENDBOT - TBOT). See Fig. S12 for model illustration and meanings of parameters.

		∂a∂i		fastsimcoal2	
	**Parameter**	**Estimate**	**95% CI**	**Estimate**	**95% CI**
		1Epoch			
ENP	N_{ANC}	18053	NA	18128	18110 – 18145
GOC	N_{ANC}	8967	NA	8988	8972 – 9003
		2Epoch			
ENP	N_{ANC}	16357	15623 – 17092	16429	16375 – 16483
	N_{CUR}	21225	20681 – 21768	21245	21126 – 21363
	T	6731	5460 – 8002	6776	6361 – 7191
GOC	N_{ANC}	14974	843 – 19671	26007	17779 – 34235
	N_{CUR}	5304	3950 – 9739	5400	5351 – 5449
	T	6803	1583 – 14427	15274	10053 – 20495
		3Epoch			
ENP	N_{ANC}	16479	15514 – 17443	16527	16427 – 16627
	N_{BOT}	23913	15775 – 32051	23528	22385 – 24671
	N_{CUR}	305	0 – 1137	184	0 – 404
	T_{BOT}	4422	992 – 7852	4559	3493 – 5625
	T_{CUR}	2	1.89 – 2.11	1	0 – 2
GOC	N_{ANC}	39098	0 – 381794	26095	17045 – 35145
	N_{BOT}	6004	0 – 536	6005	4942 – 7068
	N_{CUR}	12	0 – 1071	2317	725 – 3908
	T_{BOT}	24059	0 – 744825	18058	5730 – 30386
	T_{CUR}	0.2	0 – 19	74	0 – 3142
		3EpochTcur2			
ENP	N_{ANC}	16481	15495 – 17467	16576	16522 – 16630
	N_{BOT}	23921	22462 – 25380	23704	23139 – 24269
	N_{CUR}	306	167 – 446	348	277 – 419
	T_{BOT}	4402	3292 – 5512	4352	3969 – 4735
	T_{CUR}	2 (Fixed)	NA	2 (Fixed)	NA
		3EpochTcur3			

ENP	N_{ANC}	16481	15493 – 17469	16581	16527 – 16634
	N_{BOT}	23922	22548 – 25297	23654	23142 – 24166
	N_{CUR}	460	269 – 650	536	442 – 629
	T_{BOT}	4407	3291 – 5523	4275	3966 – 4584
	T_{CUR}	3 (Fixed)	NA	3 (Fixed)	NA
			4Epoch		
ENP	N_{ANC}	2142872	170393 – 425039	16602	16541 – 16662
	N_{BOT}	25482	0 – 10884	10396	6167 – 14624
	N_{REC}	2655	0 – 20629	18490	17594 – 19385
	N_{CUR}	19920	0 – 16181	401	126 – 676
	T_{BOT}	437505	0 – 1029835	0	0 – 0
	T_{REC}	629392	44768 – 138007	3460	2781 – 4139
	T_{CUR}	72201	0 – 113117	0	0 – 0
GOC	N_{ANC}	15031	11038 – 19023	16921	9506 – 24335
	N_{BOT}	9	0 – 43	2977	393 – 5561
	N_{REC}	51119	30175 – 77392	10150	1298 – 19001
	N_{CUR}	99	21 – 216	879	0 – 2545
	T_{BOT}	7	0 – 29	3493	0 – 11252
	T_{REC}	2362	1529 – 3342	2042	0 – 6550
	T_{CUR}	12	4 – 24	53	0 – 2193

Table S8. ∂a∂i parameter estimation for the ENP population 3-epoch model under different optimization methods. To confirm our finding of a very recent population reduction in the Eastern North Pacific population, we ran the 3-epoch model in ∂a∂i with three different optimization methods which explore the parameter space differently. In general, and according to ∂a∂i's manual (https://dadi.readthedocs.io/en/latest/api/dadi/Inference.html#dadi.Inference.optimize_cons), the optimize_log_lbfgsb and optimize_log_fmin optimization methods explore the parameter space more widely, while the optimize_log method explores it more narrowly but performs better when it starts closer to the optimum. It can be observed that all optimization methods detect very similar demographic histories for the Eastern North Pacific population, particularly the detection of a very recent (0.1 – 1.97 generations ago) and dramatic population reduction (16 – 303 effective individuals) that may be explained by the intense whaling this population suffered during the 20th century. These findings confirm that it is possible to detect very recent population reductions from whole-genome sequencing data from contemporary samples. For population size parameters (N_{ANC}, N_{BOT}, N_{CUR}), all values are in units of numbers of effective diploids. For time parameters (T_{BOT}, T_{CUR}), all values are in units of generations. N_{ANC}: Ancestral size, N_{BOT}: size after the first demographic event (expansion), N_{CUR}: size after the latest demographic event (reduction), T_{BOT}: time of the expansion, T_{CUR}: time of the recent reduction.

	**Optimization methods**		
Parameters	Optimize_log	Optimize_log_fmin	Optimize_log_lbfgsb
N_{ANC}	16,479	16,481	16,386
N_{BOT}	23,906	23,935	21,530
N_{CUR}	303	16	176
T_{BOT}	4,419	4,401	6,470
T_{CUR}	1.97	0.10	0.26
Log-likelihood	-187.4	-187.3	-275.1

Table S9. Performance of the demographic models estimated under the SFS without genotype filters for both, ∂a∂i and fastsimcoal2. K: the number of estimated parameters used in the corresponding model. Data Log-likelihood: the best possible log-likelihood of the empirical site-frequency spectrum (SFS) fit to itself. Log-likelihood: the best log-likelihood obtained for each model, measuring the fit of the model to the empirical data, and thus the performance of the model. AIC: the Akaike information criterion. Convergence of parameters is also reported.

		∂a∂i					fastsimcoal2				
**Population**	**Model**	**K**	**Data Log-likelihood**	**Log-likelihood**	**AIC**	**Converged**	**K**	**Data Log-likelihood**	**Log-likelihood**	**AIC**	**Converged**
ENP	1Epoch	0	-148.79	-15775.94	31551.88	Yes	1	-13600558.40	-13607127.63	27214257.27	No
ENP	2Epoch	2	-148.79	-236.84	477.68	Yes	3	-13600558.40	-13600587.71	27201181.44	Yes
ENP	3Epoch	4	-148.79	-206.05	420.11	Yes	5	-13600558.40	-13600574.26	27201158.53	Yes
ENP	3EpochTcur1	3	-148.79	-206.06	418.13	Yes	4	-13600558.40	-13600575.71	27201159.41	Yes
ENP	3EpochTcur2	3	-148.79	-206.09	418.18	Yes	4	-13600558.40	-13600574.14	27201156.28	Yes
ENP	3EpochTcur3	3	-148.79	-206.12	418..24	Yes	4	-13600558.40	-13600575.01	27201158.01	Yes
ENP	4Epoch	6	-148.79	-2617.87	5247.75	Yes	7	-13600558.40	-13600581.82	27201177.63	Yes

Table S10. Demographic parameters estimated using the SFS without genotype filtering for the single-population ENP models. The 95% confidence intervals for the estimated parameters are shown. If a model contains fixed parameters, it is denoted as "value (Fixed)" in the estimate column. For population size parameters (N_{ANC}, N_{BOT}, etc.), all values are in units of number of diploids. The time parameters (T, T_{CUR}, etc.), are in units of generations. Time parameter values estimated with fastsimcoal2 are converted to time intervals forward in time for better comparisons with ∂a∂i output.

		**∂a∂i**		**fastsimcoal2**	
	**Parameter**	**Estimate**	**95% CI**	**Estimate**	**95% CI**
		1Epoch			
ENP	N_{ANC}	18634	NA	18627	18602 – 18651
		2Epoch			
ENP	N_{ANC}	16835	15757 – 17912	16904	16848 – 16960
	N_{CUR}	22948	22332 – 23563	22896	22749 – 23043
	T	5381	2115 – 8647	5404	5103 – 5705
		3Epoch			
ENP	N_{ANC}	16888	15954 – 17822	16894	16838– 16950
	N_{BOT}	24264	20744 – 27785	22916	22789 – 23043
	N_{CUR}	152	0 – 547	425	155 – 695
	T_{BOT}	4494	3547 – 5441	5500	5222 – 5778
	T_{CUR}	0	0.37 – 0.43	1	0 – 2
		3EpochTcur1			
ENP	N_{ANC}	16887	15836– 17939	16937	16885 – 16988
	N_{BOT}	24277	21674 – 26880	24105	23623 – 24587
	N_{CUR}	377	0 – 1100	404	253 – 554
	T_{BOT}	4491	900 – 8081	4587	4238 – 4936
	T_{CUR}	1 (Fixed)	NA	1 (Fixed)	NA
		3EpochTcur2			
ENP	N_{ANC}	16889	15848 – 17930	17006	16959 – 17053
	N_{BOT}	24273	21821 – 26724	24628	24149 – 25107
	N_{CUR}	780	0 – 2180	629	459 – 798
	T_{BOT}	4486	1014 – 7958	4217	3975 – 4459
	T_{CUR}	2 (Fixed)	NA	2 (Fixed)	NA
		3EpochTcur3			
ENP	N_{ANC}	16890	15873 – 17907	16959	16891 – 17174
	N_{BOT}	24290	21924 – 26655	24095	23365 – 24827
	N_{CUR}	1096	0 – 3022	1178	852 – 1289
	T_{BOT}	4470	1215 – 7725	4455	3970 – 47398
	T_{CUR}	3 (Fixed)	NA	3 (Fixed)	NA

Table S11. Demographic parameter estimates for the two-population models. Parameter estimates based on the analysis of the joint SFS between the Eastern North Pacific and (ENP) and Gulf of California (GOC) populations. The demographic models are identified in the blue header lines. If a model contains fixed parameters, it is denoted as "value (Fixed)" in the estimate column. The parameter estimates and 95% Confidence Intervals derived from ∂a∂i or fastsimcoal2 are reported side by side. For population size parameters (N_{ANC}, N_{ENP}, etc.), all values are in units of number of diploids. For time parameters (T, T_W, etc.), all values are in units of generations. For time parameters derived from fastsimcoal2, values are additionally converted to time intervals forward in time for better comparisons with ∂a∂i output. For migration parameters, $m_{ENP\text{->}GOC}$ is measured as the fraction of individuals each generation in the GOC population that are new migrants from ENP, and vice versa for $m_{GOC\text{->}ENP}$. See Fig. S16 for model illustration and further explanation of parameters.

	**∂a∂i**		**fastsimcoal2**	
**Parameter**	**Estimate**	**95% CI**	**Estimate**	**95% CI**
Split-NoMigration				
N_{ANC}	17414	16764 – 18063	17024	16912 – 17136
N_{ENP}	169248	0– 807378	43487	40432 – 46541
N_{GOC}	325	147 – 515	557	509 – 605
T	255	117 – 403	433	395 – 471
Split-SymmetricMigration				
N_{ANC}	17506	16857 – 18156	17820	17710 – 17930
N_{ENP}	21412	19822 – 23064	21184	20800 – 21568
N_{GOC}	954	855 – 1047	973	910 – 1035
T	1295	1115 – 1484	1334	1254 – 1414
m	1.91E-04	1.91E-04 – 1.92E-04	1.89E-04	1.74E-04 – 2.04E-04
Split-AsymmetricMigration				
N_{ANC}	16169	15465 – 16873	16205	11550 – 20860
N_{ENP}	21225	19719 – 22783	21325	20761 – 21889
N_{GOC}	115	93 – 140	122	98 – 145
T	7305	5186 – 9588	7405	0 – 29746
$m_{ENP\text{->}GOC}$	3.38E-03	2.93E-03 – 3.80E-03	3.20E-03	2.64E-03 – 3.76E-03
$m_{GOC\text{->}ENP}$	7.16E-05	6.47E-05 – 7.80E-05	6.57E-05	5.55E-05 – 7.58E-05
Split-AsymmetricMigration-ENPChangeTw2				
N_{ANC}	16274	15586 – 16962	16307	16210 – 16404
N_{ENP}	23884	21959 – 25890	22939	22170 – 23707
N_{ENP2}	400	289 – 521	517	299 – 734
N_{GOC}	56	50 – 63	78	54 – 101
T	5371	3749 – 7116	6385	5606 – 7164
T_W	2 (Fixed)	NA	2 (Fixed)	NA
$m_{ENP\text{->}GOC}$	6.99E-03	6.68E-03 – 7.28E-03	5.06E-03	3.63E-03 – 6.50E-03
$m_{GOC\text{->}ENP}$	1.32E-04	1.11E-04 – 1.51E-04	1.08E-04	8.54E-05 – 1.30E-04
AncestralSizeChange-Split-AsymmetricMigration				

N_{ANC}	16341	15638 – 17044	16398	16316 – 16480
N_{ANC2}	25207	19534 – 31220	26764	23017 – 30511
N_{ENP}	17386	13550 – 21499	18432	17151 – 19713
N_{GOC}	114	95 – 134	124	110 – 138
T_{A}	3706	1272 – 6345	3168	1990 – 4346
T_{D}	616	155 – 1115	960	322 – 1598
$m_{ENP->GOC}$	3.42E-03	3.09E-03 – 3.72E-03	3.13E-03	2.79E-03 – 3.47E-03
$m_{GOC->ENP}$	9.24E-05	7.11E-05 – 1.12E-04	9.01E-05	7.86E-05 – 1.02E-04
AncestralSizeChange-Split-Isolation-AsymmetricMigration				
N_{ANC}	16212	14845 – 17579	16308	16155 – 16460
N_{ANC2}	22646	14976 – 31377	22283	21416 – 23150
N_{ENP}	13684	0 – 32026	17375	15715 – 19034
N_{GOC}	98	80 – 117	191	134 – 248
T_{A}	5704	0 – 18644	6000	4404 – 7596
T_{D}	106	83 – 132	213	0 – 494
T_{C}	44	27 – 62	114	2 – 226
$m_{ENP->GOC}$	4.54E-03	4.42E-03 – 4.67E-03	2.2E-03	1.67E-03 – 2.73E-03
$m_{GOC->ENP}$	3.06E-04	0 – 7.07E-04	1.99E-04	1.38E-04 – 2.59E-04
AncestralSizeChange-Split-AsymmetricMigration-GOCChange				
N_{ANC}	16320	15513 – 17128	16349	16255 – 16442
N_{ANC2}	23624	18204 – 24486	25820	14822 – 36818
N_{ENP}	11284	0 – 25684	19891	19044 – 20738
N_{GOC}	60	0 – 141	411	0 – 1236
N_{GOC2}	144	12 – 288	127	112 – 142
T_{A}	4663	523 – 9210	3099	240 – 5958
T_{D}	91	0– 246	880	0 – 3127
T_{C}	33	0 – 75	1127	388 – 1866
$m_{ENP->GOC}$	3.36E-03	3.53E-04 – 6.07E-03	3.06E-03	2.69E-03 – 3.43E-03
$m_{GOC->ENP}$	1.74E-04	0 – 3.37E-04	6.76E-05	5.97E-05 – 7.55E-05

Table S12. Likelihood ratio test (LRT) between nested two-population models. The LRT values and their significance are shown. The results for the comparison between nested ∂a∂i models are shown below the diagonal and the results for fastsimcoal2 nested model comparison are shown above the diagonal. For both demographic inference programs, the ancestral size change models (AncestralSizeChange-) have significantly better likelihoods than the split and asymmetric migration model (Split-AsymmetricMigration-). However, the parameters estimated for the more complicated ancestral size change models (AncestralSizeChange-Split-Isolation-AsymmetricMigration and AncestralSizeChange-Split-AsymmetricMigration-GOCChange) are not consistent between ∂a∂i and fastsimcoal2 or lack good convergence (see Tables S5, S11; Supplemental Results), which could indicate an overparameterization of these models. Therefore, the simpler model showing an ancestral size change event before population divergence and sustained asymmetric migration between the ENP and GOC populations (AncestralSizeChange-Split-AsymmetricMigration) seems to best represent our data. The LRT significance was evaluated with a two-sided chi-square test (χ^2) without multiple comparison adjustment with one or two degrees of freedom, depending on the number of parameter differences between models (χ^2 test for ∂a∂i models: $p_{Split\text{-}NoMigration\_vs\_Split\text{-}SymmetricMigration} = 0$, $p_{Split\text{-}SymmetricMigration\_vs\_Split\text{-}AsymmetricMigration} = 0$, $p_{Split\text{-}AsymmetricMigration\_vs\_Split\text{-}AsymmetricMigration\text{-}ENPChangeTw2} = 2.45e\text{-}72$, $p_{Split\text{-}AsymmetricMigration\_vs\_AncestralSizeChange\text{-}Split\text{-}AsymmetricMigration} = 1.01e\text{-}78$, $p_{AncestralSizeChange\text{-}Split\text{-}AsymmetricMigration\_vs\_AncestralSizeChange\text{-}Split\text{-}Isolation\text{-}AsymmetricMigration} = 0$, $p_{AncestralSizeChange\text{-}Split\text{-}AsymmetricMigration\_vs\_AncestralSizeChange\text{-}Split\text{-}AsymmetricMigration\text{-}GOCChange} = 0$. χ^2 test for fastsimcoal2 models: $p_{Split\text{-}NoMigration\_vs\_Split\text{-}SymmetricMigration} = 0$, $p_{Split\text{-}SymmetricMigration\_vs\_Split\text{-}AsymmetricMigration} = 0$, $p_{Split\text{-}AsymmetricMigration\_vs\_Split\text{-}AsymmetricMigration\text{-}ENPChangeTw2} = 2.97e\text{-}35$, $p_{Split\text{-}AsymmetricMigration\_vs\_AncestralSizeChange\text{-}Split\text{-}AsymmetricMigration} = 1.35e\text{-}36$, $p_{AncestralSizeChange\text{-}Split\text{-}AsymmetricMigration\_vs\_AncestralSizeChange\text{-}Split\text{-}Isolation\text{-}AsymmetricMigration} = 5.43e\text{-}101$, $p_{AncestralSizeChange\text{-}Split\text{-}AsymmetricMigration\_vs\_AncestralSizeChange\text{-}Split\text{-}AsymmetricMigration\text{-}GOCChange} = 0.03$). NA denotes the LRT cannot be performed because the models are not nested. Significance level: * $p < 0.05$, ** $p < 1.0e\text{-}25$, *** $p < 1.0e\text{-}50$.

	Split-NoMigration	Split-SymmetricMigration	Split-AsymmetricMigration	Split-AsymmetricMigration-ENPChangeTw2	AncestralSizeChange-Split-AsymmetricMigration	AncestralSizeChange-Split-Isolation-AsymmetricMigration	AncestralSizeChange-Split-AsymmetricMigration-GOCChange
Split-NoMigration	–	47700.22***					
Split-SymmetricMigration	104491.5***	–	48798.02***				
Split-AsymmetricMigration		113413.4***	–	153.5**	165.18**		
Split-AsymmetricMigration-ENPChangeTw2			323.54***	–	NA		
AncestralSizeChange-Split-AsymmetricMigration			352.86***	NA	–	455.16***	6.62*
AncestralSizeChange-Split-Isolation-AsymmetricMigration					2012.76***	–	NA
AncestralSizeChange-Split-AsymmetricMigration-GOCChange					2211.46***	NA	–

Table S13. Summary of the ghost population demographic models' performances. The populations are ENP-GOC. *K:* the number of estimated parameters used in the corresponding model. Data Log-likelihood: the best possible log-likelihood of the empirical site-frequency spectrum (SFS) fit to itself. Log-likelihood: the best log-likelihood obtained for each particular model, measuring the fit of the model to the empirical data, and thus the performance of the model. AIC: the Akaike information criterion is calculated using the equation AIC = 2*K - 2*Log-likelihood. Convergence of parameters is also reported. See Methods for full descriptions of the models and Fig. S18 for model illustration.

	**∂a∂i**					**fastsimcoal2**				
**Model**	**K**	**Data Log-likelihood**	**Log-likelihood**	**AIC**	**Converged**	**K**	**Data Log-likelihood**	**Log-likelihood**	**AIC**	**Converged**
GhostPopSplit-ENPGOCSplit-AsymmetricMigration (WNP)	8	-3314.79	-6215.16	12446.33	Yes	9	-15378118	-15379400	30758819	Yes
GhostPopSplit-ENPGOCSplit-AsymmetricMigration (SP)	N/A	N/A	N/A	N/A	N/A	9	-15378118	-15379637	30759292	No

Table S14. Demographic parameter estimates for the two-population models including ghost populations. Results for two ghost demographic models are presented, one with the ghost population model representing the Western North Pacific (GhostPopSplit-ENPGOCSplit-AsymmetricMigration (WNP)) and the other with the ghost population representing the South Pacific (GhostPopSplit-ENPGOCSplit-AsymmetricMigration (SP)). The 95% confidence intervals for the estimated parameters are shown. For population size parameters all values are in units of number of diploids. The time parameters are in units of generations. Time parameter values estimated with fastsimcoal2 are converted to time intervals forward in time for better comparisons with ∂a∂i output. We only estimated one ghost model (GhostPopSplit-ENPGOCSplit-AsymmetricMigration (WNP)) with ∂a∂i (See Methods). N_{ANC} is the size of the ancestral populations before the split between the ghost population and the ancestral population of the Eastern North Pacific, $N_{ANC-ENP}$ is the size of the ancestral Eastern North Pacific population that diverged from the ghost population, N_{ENP} is the size of the current Eastern North Pacific population, N_{GOC} is the size of the current Gulf of California population, T_{D-ENP} is divergence time between the ghost and ancestral Eastern North Pacific populations, T_{D-GOC} is the divergence time between the Eastern North Pacific and Gulf of California populations, $m_{ENP->GOC}$ is the migration rate from the Eastern North Pacific to the Gulf of California population, $m_{GOC->ENP}$ is the migration rate from the Gulf of California population to the Eastern North Pacific population, $m_{Ghost->GOC}$ is the migration rate from the ghost population to the Gulf of California. For a graphic representation of the ghost population model please see Fig. S18.

	**∂a∂i**		**fastsimcoal2**	
**Parameter**	**Estimate**	**95% CI**	**Estimate**	**95% CI**
GhostPopSplit-ENPGOCSplit-AsymmetricMigration (WNP)				
N_{ANC}	16336	16041 – 16729	16469	16357 – 16581
$N_{ANC-ENP}$	24500	15840 – 32510	24138	6423 – 41852
N_{ENP}	14587	10832 – 19325	9997	6334 – 13660
N_{GOC}	50	23 - 92	14	0 – 56
T_{D-ENP}	4115	2459 – 8592	4190	2206 – 6174
T_{D-GOC}	297	0 - 537	107	0 – 862
$m_{ENP->GOC}$	7.55E-03	3.24E-04 – 9.73E-03	2.69E-02	0 – 5.81E-02
$m_{GOC->ENP}$	1.61E-04	4.63E-05 – 8.91E-04	3.85E-04	3.18E-05 – 7.39E-04
$M_{Ghost->GOC}$	2.09E-04	1.96E-05 – 5.72E-04	1.12E-03	0 – 3.68E-03
GhostPopSplit-ENPGOCSplit-AsymmetricMigration (SP)				
N_{ANC}	N/A	N/A	29723	12278 – 47167
$N_{ANC-ENP}$	N/A	N/A	23100	0 – 48960
N_{ENP}	N/A	N/A	18583	15328 – 21837
N_{GOC}	N/A	N/A	5	0 – 18
T_{D-ENP}	N/A	N/A	39607	0 – 106914
T_{D-GOC}	N/A	N/A	43884	13596 – 74172
$m_{ENP->GOC}$	N/A	N/A	5.7E-02	3.00E-02 – 8.40E-02
$m_{GOC->ENP}$	N/A	N/A	1.39E-03	9.08E-04 – 1.87E-03
$m_{Ghost->GOC}$	N/A	N/A	2.19E-02	3.88E-03 – 3.98E-02

Table S15. Summary statistics for putatively deleterious variation using SIFT. For the four mutation types evaluated (SYN: synonymous, TOL: tolerated nonsynonymous, DEL: deleterious nonsynonymous, LOF: loss-of-function), the normalized average number of derived alleles, heterozygous genotypes and homozygous derived genotypes are reported for each population. The difference between the ENP and GOC (ENP – GOC) populations and the percentage of difference ((ENP – GOC)/ENP) are also reported. The total number of segregating sites analyzed for each mutation type is shown (# of sites), which sum up to 116,908 total segregating sites analyzed. The P-value is for two-tailed Mann-Whitney U-tests comparing numbers of genotypes, derived alleles per individual between the ENP and GOC populations.

Mutation Type	# of sites	ENP	GOC	ENP – GOC	(ENP – GOC)/ENP	*p*-value (MWU test)
			Number of derived alleles			
SYN	59384	25600	25663	-63	-0.25%	0.078
TOL	35530	13812	13828	-16	-0.12%	0.520
DEL	20972	5864	5983	-119	-2.03%	1.20E-07
LOF	1022	376	376	0	0.03%	0.870
			Number of heterozygous genotypes			
SYN	59384	10568	6610	3958	37.45%	2.90E-12
TOL	35530	5768	3560	2209	38.29%	2.90E-12
DEL	20972	2887	1825	1062	36.79%	2.90E-12
LOF	1022	159	96	63	39.48%	2.90E-12
			Number of derived homozygous genotypes			
SYN	59384	7516	9526	-2011	-26.75%	2.90E-12
TOL	35530	4022	5134	-1112	-27.66%	2.90E-12
DEL	20972	1488	2079	-591	-39.68%	2.90E-12
LOF	1022	108	140	-31	-28.98%	2.90E-12

Table S16. Comparisons of the available genome assemblies in the *Balaenopteridae* family. We used the divergence time estimated from Árnason et al. 2018[29] and karyotypes from O'Brien et al. 2007[30]. All statistics are obtained from individual NCBI Genome Assembly summary page or compiled from the articles with genome release.

	**Fin whale (NCBI)** Baphy	**Fin whale** DNAzoo	**Fin whale (NCBI)** SBiKF_Bphy_ph2	**Minke whale**	**Blue whale**	**Rice's whale**	**Humpback whale**
**Divergence Time (Mya)**	0	0	0	10.48	8.35	8.35	4.98
**Species**	*Balaenoptera physalus*	*Balaenoptera physalus*	*Balaenoptera physalus*	*Balaenoptera acutorostrata scammoni*	*Balaenoptera musculus*	*Balaenoptera ricei*	*Megaptera novaeangliae*
**Karyotype**	2n = 44	2n = 44	2n = 44	2n = 44	2n = 44	2n = 44	2n = 44
**Last updated**	10/1/19	11/9/21	05/11/22	10/31/13	10/29/20	11/8/19	3/6/19
**Reference**	Westbury et al. 2019[31]	Dudchenko et al. 2017[32]	Wolf et al. 2022[9]	Yim et al. 2014[33]	Rhie et al. 2020[34]	Dudchenko et al. 2017[32]	Tollis et al. 2019[35]
**NCBI Accession / DNAzoo release**	*GCA_008795845.1* (https://www.ncbi.nlm.nih.gov/assembly/GCA_008795845.1/)	*DNAzoo_fin_whale* (https://dnazoo.s3.wasabisys.com/index.html?prefix=Balaenoptera_physalus/)	*GCA_023338255.1* (https://www.ncbi.nlm.nih.gov/assembly/GCA_023338255.1)	*GCF_000493695.1* (https://www.ncbi.nlm.nih.gov/assembly/GCF_000493695.1/)	*GCF_009873245.2* (https://www.ncbi.nlm.nih.gov/assembly/GCF_009873245.2/)	*DNAzoo_Rice's_whale* (https://dnazoo.s3.wasabisys.com/index.html?prefix=Balaenoptera_ricei/)	*GCA_004329385.1* (https://www.ncbi.nlm.nih.gov/assembly/GCA_004329385.1/)
**Sequencing method**	Illumina	Illumina + HiC	10X Chromium	Illumina HiSeq 2000	PacBio Sequel I; Illumina NovaSeq; 10X Genomics chromium; Dovetail Genomics HiC	Illumina + HiC	Illumina HiSeq
**Assembly method**	SOAPdenovo v. 2; Cross-species scaffolding v. June-2018	w2rap + 3D Assembly + Juicebox Assembly Tools	supernova v. 2.1.1	SOAPdenovo v. 16-Mar-2012	VGP standard assembly pipeline v. 1.5	w2rap + 3D Assembly + Juicebox Assembly Tools	Meraculous + HiRise v. Feb-2016
**Assembly level**	Scaffold	Chromosome-length scaffold	Scaffold	Scaffold	Chromosome	Chromosome-length scaffold	Scaffold
**Raw coverage**	40.0x	N/A	30.0x	92x	51.16x	N/A	102.0x
**Total length (bp)**	2.46E+09	2.74E+09	2.41E+09	2.43E+09	2.37E+09	2.38E+09	2.27E+09
**Number of scaffolds**	62302	1361899	13140	10776	106	141314	2558
**Scaffold N50 (bp)**	8.71E+05	7.77E+07	2.49E+07	1.28E+07	1.10E+08	9.96E+07	9.14E+06

**Contig N50 (bp)**	4.49E+03	3.89E+04	1.46E+05	2.27E+04	6.32E+06	7.12E+04	1.23E+04
**Annotation method**	MAKER2	MAKER2	MAKERv2.31 + INTERPROSCAN v5	NCBI Eukaryotic Genome Annotation Pipeline	NCBI Eukaryotic Genome Annotation Pipeline	MAKER2	MAKER2
**Genes and pseudogenes**	2.02E+04	N/A	1.73E+04 (transcripts)	2.68E+04	2.72E+04	2.75E+04	N/A
**protein-coding genes**	1.97E+04	N/A	N/A	1.88E+04	1.97E+04	2.75E+04	2.41E+04
**CDS**	1.97E+04	N/A	N/A	3.77E+04	5.23E+04	1.69E+05	N/A

Table S17. Number of sites that passed all filters in our genotype pipeline for each analysis mentioned in the main text. Unless specifically mentioned, all the analyses used the all50 dataset.

Dataset	Samples	Filtration method	Analyses in which the dataset was used	Number of sites that passed all filters
**all50**	All 50 fin whales	Standard	All the analyses except those mentioned for the other datasets below.	890,858,824
**f50b4**	All 50 fin whales + four Mysticeti species	Standard	Genome wide heterozygosity for four Mysticeti species (Fig.2 and Fig. S8). Neighbor-joining tree (Fig. S5).	880,177,286
**genotype-filter-free**	All 50 fin whales	No genotype filters	Demographic inference using the SFS without genotype filtering (Table S10).	934,524,879
**10-fin-ref**	Subset of 10 samples (five per population; see Supplemental Methods)	Standard	Complementary analyses of genome-wide heterozygosity and total ROH length using the fin whale reference genome (Table S2; Figure S1)	1,084,268,877

Supplementary References

1. McCoy, R. C., Garud, N. R., Kelley, J. L., Boggs, C. L. & Petrov, D. A. Genomic inference accurately predicts the timing and severity of a recent bottleneck in a nonmodel insect population. *Molecular Ecology* **23**, 136–150 (2014).
2. Beichman, A. C. *et al.* Genomic analyses reveal range-wide devastation of sea otter populations. *Molecular Ecology* mec.16334 (2022).
3. Simão, F. A., Waterhouse, R. M., Ioannidis, P., Kriventseva, E. V. & Zdobnov, E. M. BUSCO: assessing genome assembly and annotation completeness with single-copy orthologs. *Bioinformatics* **31**, 3210–3212 (2015).
4. Manni, M., Berkeley, M. R., Seppey, M., Simão, F. A. & Zdobnov, E. M. BUSCO Update: Novel and Streamlined Workflows along with Broader and Deeper Phylogenetic Coverage for Scoring of Eukaryotic, Prokaryotic, and Viral Genomes. *Molecular Biology and Evolution* **38**, 4647–4654 (2021).
5. Rocha, R. C., Clapham, P. J. & Ivashchenko, Y. V. Emptying the oceans: a summary of industrial whaling catches in the 20th century. *Marine Fisheries Review* **76**, 37–48 (2014).
6. Mizroch, S. A., Rice, D. W., Zwiefelhofer, D., Waite, J. & Perryman, W. L. Distribution and movements of fin whales in the North Pacific Ocean. *Mammal Review* **39**, 193–227 (2009).
7. Arguello, J. R., Laurent, S. & Clark, A. G. Demographic History of the Human Commensal Drosophila melanogaster. *Genome Biology and Evolution* **11**, 844–854 (2019).
8. Rivera-León, V. E. *et al.* Long-term isolation at a low effective population size greatly reduced genetic diversity in Gulf of California fin whales. *Scientific Reports* **9**, 12391 (2019).
9. Wolf, M., de Jong, M., Halldórsson, S. D., Árnason, Ú. & Janke, A. Genomic Impact of Whaling in North Atlantic Fin Whales. *Molecular Biology and Evolution* **39**, msac094 (2022).
10. Pérez-Álvarez, Mj. *et al.* Contrasting phylogeographic patterns among Northern and Southern Hemisphere fin whale populations with new data from the Southern Pacific. *Front. Mar. Sci.* **8**, (2021).
11. Beichman, A. C., Huerta-Sanchez, E. & Lohmueller, K. E. Using genomic data to infer historic population dynamics of nonmodel organisms. *Annual Review of Ecology, Evolution, and Systematics* (2018).
12. Nielsen, R., Paul, J. S., Albrechtsen, A. & Song, Y. S. Genotype and SNP calling from next-generation sequencing data. *Nat Rev Genet* **12**, 443–451 (2011).
13. Han, E., Sinsheimer, J. S. & Novembre, J. Characterizing Bias in Population Genetic Inferences from Low-Coverage Sequencing Data. *Molecular Biology and Evolution* **31**, 723–735 (2014).
14. Xue, Y. *et al.* Mountain gorilla genomes reveal the impact of long-term population decline and inbreeding. *Science* **348**, 242–245 (2015).
15. Abascal, F. *et al.* Extreme genomic erosion after recurrent demographic bottlenecks in the highly endangered Iberian lynx. *Genome Biol* **17**, 251 (2016).
16. Armstrong, E. E. *et al.* Recent Evolutionary History of Tigers Highlights Contrasting Roles of Genetic Drift and Selection. *Molecular Biology and Evolution* **38**, 2366–2379 (2021).
17. Robinson, J. A. *et al.* Genomic signatures of extensive inbreeding in Isle Royale wolves, a population on the threshold of extinction. *Science Advances* **5**, eaau0757 (2019).
18. Robinson, J. A. *et al.* The critically endangered vaquita is not doomed to extinction by inbreeding depression. *Science* **376**, 635–639 (2022).

19. Gutenkunst, R. N., Hernandez, R. D., Williamson, S. H. & Bustamante, C. D. Inferring the joint demographic history of multiple populations from multidimensional SNP frequency data. *PLoS genet* **5**, e1000695 (2009).
20. Städler, T., Haubold, B., Merino, C., Stephan, W. & Pfaffelhuber, P. The Impact of Sampling Schemes on the Site Frequency Spectrum in Nonequilibrium Subdivided Populations. *Genetics* **182**, 205–216 (2009).
21. Peter, B. M., Wegmann, D. & Excoffier, L. Distinguishing between population bottleneck and population subdivision by a Bayesian model choice procedure. *Molecular Ecology* **19**, 4648–4660 (2010).
22. Heller, R., Chikhi, L. & Siegismund, H. R. The Confounding Effect of Population Structure on Bayesian Skyline Plot Inferences of Demographic History. *PLOS ONE* **8**, e62992 (2013).
23. Mazet, O., Rodríguez, W., Grusea, S., Boitard, S. & Chikhi, L. On the importance of being structured: instantaneous coalescence rates and human evolution—lessons for ancestral population size inference? *Heredity* **116**, 362–371 (2016).
24. Chikhi, L. *et al.* The IICR (inverse instantaneous coalescence rate) as a summary of genomic diversity: insights into demographic inference and model choice. *Heredity* **120**, 13–24 (2018).
25. Alter, S. E., Rynes, E. & Palumbi, S. R. DNA evidence for historic population size and past ecosystem impacts of gray whales. *Proc. Natl. Acad. Sci. U.S.A.* **104**, 15162–15167 (2007).
26. Beerli, P. Effect of unsampled populations on the estimation of population sizes and migration rates between sampled populations. *Molecular Ecology* **13**, 827–836 (2004).
27. Waples, R. S. *et al.* Guidelines for genetic data analysis. *Journal of Cetacean Research and Management* **18**, 33–80 (2018).
28. Morgulis, A., Gertz, E. M., Schäffer, A. A. & Agarwala, R. WindowMasker: window-based masker for sequenced genomes. *Bioinformatics (Oxford, England)* **22**, 134–141 (2006).
29. Árnason, Ú., Lammers, F., Kumar, V., Nilsson, M. A. & Janke, A. Whole-genome sequencing of the blue whale and other rorquals finds signatures for introgressive gene flow. *Science Advances* **4**, eaap9873 (2018).
30. O'Brien, S. J., Menninger, J. C. & Nash, W. G. *Atlas of Mammalian Chromosomes*. (2007).
31. Westbury, M. V., Petersen, B. & Lorenzen, E. D. Genomic analyses reveal an absence of contemporary introgressive admixture between fin whales and blue whales, despite known hybrids. *PLoS ONE* **14**, e0222004 (2019).
32. Dudchenko, O. *et al.* De novo assembly of the *Aedes aegypti* genome using Hi-C yields chromosome-length scaffolds. *Science* **356**, 92–95 (2017).
33. Yim, H.-S. *et al.* Minke whale genome and aquatic adaptation in cetaceans. *Nature Genetics* **46**, 88–92 (2014).
34. Rhie, A. *et al.* Towards complete and error-free genome assemblies of all vertebrate species. *Nature* **592**, 737–746 (2021).
35. Tollis, M. *et al.* Return to the Sea, Get Huge, Beat Cancer: An Analysis of Cetacean Genomes Including an Assembly for the Humpback Whale (Megaptera novaeangliae). *Mol Biol Evol* **36**, 1746–1763 (2019).
