# Supplementary material for: The genomic footprint of whaling and isolation in fin whale populations
Source: Nat Commun. 2023 Sep 12;14:5465. doi: 10.1038/s41467-023-40052-z (PMC10497599; doi:10.1038/s41467-023-40052-z)
Supplement: Supplementary file 3 — Reporting Summary [file 41467_2023_40052_MOESM3_ESM.pdf]

nature portfolio

Corresponding author(s): Sergio F. Nigenda-Morales, Meixi Lin, Andres Moreno-Estrada, Robert Wayne

Last updated by author(s): 2023/06/30

Reporting Summary

Nature Portfolio wishes to improve the reproducibility of the work that we publish. This form provides structure for consistency and transparency in reporting. For further information on Nature Portfolio policies, see our Editorial Policies and the Editorial Policy Checklist.

Statistics

For all statistical analyses, confirm that the following items are present in the figure legend, table legend, main text, or Methods section.

n/a	Confirmed	
☐	☒	The exact sample size (n) for each experimental group/condition, given as a discrete number and unit of measurement
☐	☒	A statement on whether measurements were taken from distinct samples or whether the same sample was measured repeatedly
☐	☒	The statistical test(s) used AND whether they are one- or two-sided<br>*Only common tests should be described solely by name; describe more complex techniques in the Methods section.*
☐	☒	A description of all covariates tested
☐	☒	A description of any assumptions or corrections, such as tests of normality and adjustment for multiple comparisons
☐	☒	A full description of the statistical parameters including central tendency (e.g. means) or other basic estimates (e.g. regression coefficient) AND variation (e.g. standard deviation) or associated estimates of uncertainty (e.g. confidence intervals)
☐	☒	For null hypothesis testing, the test statistic (e.g. F, t, r) with confidence intervals, effect sizes, degrees of freedom and P value noted<br>*Give P values as exact values whenever suitable.*
☒	☐	For Bayesian analysis, information on the choice of priors and Markov chain Monte Carlo settings
☒	☐	For hierarchical and complex designs, identification of the appropriate level for tests and full reporting of outcomes
☒	☐	Estimates of effect sizes (e.g. Cohen's d, Pearson's r), indicating how they were calculated

Our web collection on statistics for biologists contains articles on many of the points above.

Software and code

Policy information about availability of computer code

Data collection	No software was used during field collection and DNA extraction. During generation of vcf files, the following software was used:<br>FastQC v.0.11.8<br>BWA-MEM v.0.7.17<br>QUALIMAP v.2.2<br>GATK v.3.8<br>snpEff v.4.3.1<br>SIFT4G v.6.0<br>BLAST v.2.7.1<br>picard v.2.20.3<br>WindowMasker v.2.2.22<br>RepeatMasker v.4.1.0<br><br>Custom scripts are publicly available on Zenodo: DOI: 10.5281/zenodo.7980107 (https://doi.org/10.5281/zenodo.7980107)
Data analysis	The following R packages and libraries were used in R v.3.6.2:<br>SNPRelate v.1.16.0<br>SeqArray v.1.26.2<br>gdsfmt v.1.22.0

ape v.5.3
RZooRoH v.0.2.3
vcfR v.1.12.0
ggtree v.2.0.4
ggOceanMaps v.0.4.3

The package dadi v.2.2.1 was used with python 2.7.15

Other software and code used in the analyses:
bcftools v.1.9
bedtools v.2.28.0
PLINK v.1.90
ADMIXTURE V.1.3.0
fastsimcoal2 v.2.6
samtools v.1.9
msprime v.0.7.4
SLiM v.3.3.2
easySFS v.0.0.1 https://github.com/isaacovercast/easySFS

Custom scripts are publicly available on Zenodo: DOI: 10.5281/zenodo.7980107 (https://doi.org/10.5281/zenodo.7980107)

For manuscripts utilizing custom algorithms or software that are central to the research but not yet described in published literature, software must be made available to editors and reviewers. We strongly encourage code deposition in a community repository (e.g. GitHub). See the Nature Portfolio guidelines for submitting code & software for further information.

Data

Policy information about availability of data

All manuscripts must include a data availability statement. This statement should provide the following information, where applicable:

- Accession codes, unique identifiers, or web links for publicly available datasets
- A description of any restrictions on data availability
- For clinical datasets or third party data, please ensure that the statement adheres to our policy

The raw sequence data generated in this study is deposited in NCBI's Sequence Read Archive (SRA) database under accession numbers SRR23615109 - SRR23615158 (BioSample SAMN33439338 - SAMN33439387; BioProject PRJNA938516 [https://www.ncbi.nlm.nih.gov/bioproject/938516]; see Table S1 for details). The sequence data for the additional mysticete species used in this study are available in NCBI's SRA database under accession numbers SRR5665640 (https://www.ncbi.nlm.nih.gov/sra/SRR5665640), SRR1802584 (https://www.ncbi.nlm.nih.gov/sra/SRR1802584), SRR5665644 (https://www.ncbi.nlm.nih.gov/sra/SRR5665644) and SRR5665639 (https://www.ncbi.nlm.nih.gov/sra/SRR5665639), please see Table S1 for details. The cpg island data are available in the UCSC genome browser (http://hgdownload.soe.ucsc.edu/goldenPath/balAcu1/database/). The balenopterid genomes assemblies used for the comparison shown in Table S16 are available in NCBI's Assembly database under accession numbers GCA_008795845.1 (https://www.ncbi.nlm.nih.gov/assembly/GCA_008795845.1/), GCA_023338255.1 (https://www.ncbi.nlm.nih.gov/assembly/GCA_023338255.1/), GCF_000493695.1 (https://www.ncbi.nlm.nih.gov/assembly/GCF_000493695.1/), GCF_009873245.2 (https://www.ncbi.nlm.nih.gov/assembly/GCF_009873245.2/), GCA_004329385.1 (https://www.ncbi.nlm.nih.gov/assembly/GCA_004329385.1/), or in the DNA Zoo database under accession names Balaenoptera_physalus (https://dnazoo.s3.wasabisys.com/index.html?prefix=Balaenoptera_physalus/) and Balaenoptera_ricei (https://dnazoo.s3.wasabisys.com/index.html?prefix=Balaenoptera_ricei/). Source data are provided with this paper.

Human research participants

Policy information about studies involving human research participants and Sex and Gender in Research.

Reporting on sex and gender	No humans were used in this study.
Population characteristics	N/A
Recruitment	N/A
Ethics oversight	N/A

Note that full information on the approval of the study protocol must also be provided in the manuscript.

Field-specific reporting

Please select the one below that is the best fit for your research. If you are not sure, read the appropriate sections before making your selection.

☐ Life sciences ☐ Behavioural & social sciences ☒ Ecological, evolutionary & environmental sciences

For a reference copy of the document with all sections, see nature.com/documents/nr-reporting-summary-flat.pdf

Ecological, evolutionary & environmental sciences study design

All studies must disclose on these points even when the disclosure is negative.

Study description	This study comprises whole genome resequencing of 50 samples of fin whales, genomic diversity analyses, demographic inferences, estimation of deleterious variation and simulations of deleterious variation and genetic load.
Research sample	The research sample consist of 50 fin whale (Balaenoptera physalus) individuals from two different populations. For all this individuals biopsy punches were taken, DNA was extracted and whole genome resequencing was performed. These samples were collected between 1995 and 2017. We aimed to sample at least 20 individuals per population. We were able to sample 30 individuals from the Eastern North Pacific population and 20 for the Gulf of California population. The rationale behind selecting these populations for our research is that the Eastern North Pacific population was severely depleted by whaling activities during the 20th century, whereas the Gulf of California population has been small and isolated for several generations. These differences in demographic history will allow us to explore the genomic consequences of different types of population reductions.<br>The geographic distribution of the samples obtained in the Eastern North Pacific population is as follows: California (9), Oregon (4), Washington (2), British Columbia (3) and Alaska (12). The geographic distribution for the samples obtained in the Gulf of California is as follows: Bahía de La Paz (3), Bahía de Loreto (6), Bahía de los Angeles (5), Bahía Kino (3), North of Tiburon Island (1), Puerto Refugio (1) and out of Bahía Los Frailes (1). The 30 samples taken in locations of the Eastern North Pacific intends to represent the Eastern North Pacific population, while the samples taken in locations within the Gulf of California intends to represent the Gulf of California population.<br>We sampled 20 to 30 individuals from each population because this should be enough to reach statistically significant results, specially when whole genome data at relatively high coverage (27x) is analyzed.<br>The determination of age and sex were not relevant for our study. However, we have sex information, determine by PCR amplification, for the 30 Eastern North Pacific individuals. Of those, 50% were males and 50% females.<br>Since a high-quality reference genome for the fin whale does not exist, we used the Minke whale genome as reference. This genome is deposited in the NCBI database (BalAcu1.0, Assembly GCF_000493695.1).<br>For some genomic variation comparative analysis, we used existing whole genome sequence data of four baleen whale species. The data was downloaded from NCBI's Sequence Read Archive (SRA) database. The accession numbers of these sequences data are shown (in parenthesis): Eubalaena glacialis (SRR5665640), Balaenoptera acutorostrata (SRR1802584), Balaenoptera musculus (SRR5665644), Megaptera novaeangliae (SRR5665639).
Sampling strategy	The tissues used in this project are small skin biopsies that were collected at sea from a small boat using stainless steel biopsy darts. Briefly, once a whale was observed the boat approached slowly and no closer than 20 meters (22 yards). When the whale emerged to breath the stainless-steel modified dart was deployed using a biopsy rifle or crossbow to take the skin sample of the dorsal part of the animal close to the dorsal fin. Once the dart is observed to be on target the boat waited for the whale to go away and then approached to retrieve the biopsy dart that was floating. Then, the biopsy is preserved in an ethanol solution at 80 - 90% of concentration. The biopsy dimensions are usually 4 millimeters of diameter and 3 centimeters long. Before deployment the stainless-steal biopsy dart was sterilized using ethanol with a concentration of 90%. This protocol only takes a small skin sample and the animal is not harassed for long periods of time.<br>No statistical methods were used to predetermine sample size. The sample size was selected before analysis was begun based on available samples and budgetary constraints for sequencing. We sought to include 20 samples per population, which based on the literature of non-model organisms might be sufficient to obtain power for statistical comparisons.
Data collection	The 50 fin whales' tissue samples used in this study were previously collected during field work for research on other projects. The tissue samples were obtained following a standard protocol to obtain biopsies from free-ranging cetacean species using a biopsy riffle and a stainless-steel modified dart. The authors S. F. Nigenda-Morales and A. C. Beichman coordinated for DNA extractions and library preparations. DNA extraction was performed using the QIAGEN QIAamp DNA Mini Kit (Qiagen; California, USA). The genomic libraries were prepared from extracted DNA using the Illumina TruSeq DNA PCR-free standard kit (Illumina; California, USA) following the manufacturer instructions. Whole genome sequencing was performed using the 150-bp paired-end protocol on Illumina HiSeqX or NovaSeq6000 platforms. Library preparation and sequencing were performed in Fulgent genetics' sequencing core facility (Fulgent genetics LLC; California, USA). The authors S. F. Nigenda-Morales and M. Lin recorded the sequencing data. The authors J. Urbán R. and L. Viloria-Gómora recorded the collection years and locations for the Gulf of California samples. The author F. I. Archer recorded the collection years and locations for the Eastern North Pacific samples.
Timing and spatial scale	As stated above, the samples utilized in this study have been compiled from multiple field trips occurring in the US and Mexico. For the timing, all samples used in this study were collected from 1995 to 2017. The frequency and periodicity of sampling is not applicable to our study since genomic resequencing data does not vary with fine scale collection time. We chose this timing of collections to only include individuals that were collected after the whaling moratoriums took effect in the 1980s. No major gap between collection periods is observed though 43 out of the 50 individuals has been collected before 2010. For the spatial scale, 30 samples were collected throughout the Eastern North Pacific (represented by individuals from the coasts of California [9], Oregon [4], Washington [2], British Columbia [3] and Alaska [12]). Then, 20 samples were collected in the Gulf of California (from seven different localities; Bahía de La Paz [3], Bahía de Loreto [6], Bahía de los Angeles [5], Bahía Kino [3], North of Tiburon Island [1], Puerto Refugio [1] and out of Bahía Los Frailes [1]).
Data exclusions	Whenever data were excluded, we describe the exclusions and the rationale in the text. Specifically, we performed data exclusions in three settings.<br>First, for the exclusion of low quality genotype calls, we used pre-established exclusion criteria recommended by the GATK best practice guideline (https://gatk.broadinstitute.org/hc/en-us/sections/360007226651-Best-Practices-Workflows). We performed a stringent set of quality and depth filters for the genotype calls, keeping only high-quality biallelic SNPs and monomorphic genotypes. For each individual, only genotypes with a minimum depth of eight reads and maximum depth of 2.5x mean depth; a minimum Phred score of 20 and expected allele balance (≥ 0.9 for homozygous reference genotypes; ≥ 0.2 & ≤ 0.8 for heterozygous genotypes and ≤ 0.1 for homozygous alternative genotypes) were kept. Each site was then filtered using the following criteria. Sites that 1) failed GATK

recommended hard filters (QD < 2.0 || FS > 60.0 || MQ < 40.0 || MQRankSum < -12.5 || ReadPosRankSum < -8.0 || SOR > 3.0), 2) had low Phred score (QUAL < 30), 3) had more than 20% missing genotypes, 4) had more than 75% heterozygous genotypes or 5) fell within repeat regions identified by WindowMasker or RepeatMasker or CpG islands identified by UCSC genome browser, were marked as failed filtration.
Second, for estimation of ROH using bcftools, three individuals (admixture proportion > 0.5: ENPCA09, GOC010; low genotype depth: ENPOR12) were excluded in bcftools ROH analyses to avoid biasing allele frequency estimations.
Third, for reconstructing demographic history and quantifying putatively deleterious variation, to avoid biases caused from low-quality data, there are pre-established exclusion criteria to remove individuals that have low genotype depth, high admixture proportion and high kinship. Therefore, we excluded six individuals (Low genotype depth: "ENPOR12"; Admixture proportion > 0.25: "ENPCA01", "ENPCA09", "GOC010"; Kinship > 0.15: "GOC080", "GOC111") in SFS projection and extraction of deleterious variants.

Reproducibility

We have taken extensive measures to verify the reproducibility of our findings.
In our study design, we aim to sample adequately within the two populations with at least 20 individuals per population and achieve higher sequence coverage (at least 20X, the actual mean coverage was 27X). In empirical analyses at the individual's level (such as the population structure, genome-wide pattern of variation, runs of homozygosity and patterns of deleterious variation), the findings observed within each population are consistent across the individuals, serving as a confirmation of the reproducibility in individual's patterns. In simulations, we ran 25 replicates of each demographic scenarios tested, the observed patterns were consistent across the replicates, confirming the reproducibility.
In our analyses, we perform the same analyses using different and well established softwares whenever possible to reproduce the findings regardless of the softwares used. For example, in the runs of homozygosity analyses, we used both bcftools and RZooRoH. In the demographic inference, we tested different models employing both coalescent (fastsimcoal2) and diffusion approximation (∂a∂i) methods. In the identification of putatively deleterious mutations, we employed two mutation impact scoring system implemented by snpEff or SIFT. The results across softwares are always reproducible.
In the demographic analyses, we included additional reproducibility tests including performing additional inference runs varying the time for the whaling reduction, using different optimization methods and performing coalescent SFS simulations to confirm our power to detect such recent decline in the single population Eastern North Pacific demographic model.
All attempts to repeat the experiments as noted above were successful. The data analysis code is publicly available here: https://doi.org/10.5281/zenodo.7980107. We will also make the raw data and important derived data necessary to reproduce the results publicly available upon acceptance. During review period, these data are available upon request.

Randomization

The individuals collected from the Eastern North Pacific and the Gulf of California were randomly subsetted from the available tissue collection archives. No future randomization is necessary given the nature and scope of population genomics data.

Blinding

Blinding during our data collection was relative because sampling was opportunistic due to the biology and behavior of free-ranging cetaceans. Although sampling efforts are made in areas where fin whales are know to be present, the encounters with fin whales individuals occur randomly. Therefore, there is blinding because it is unknown if any samples will be collected any given day and if samples are collected, the sampled individuals are unknown.
The library preparation and DNA sequencing were performed in Fulgent genetics' sequencing core facility (Fulgent genetics LLC; California, USA). Fulgent is blind to the individual's geographical origin or previous genetic knowledge.
All the rest of the genomic populations analyses performed do not require blinding because the results are not affected by knowing the identity of the analyzed individuals.

Did the study involve field work? ☒ Yes ☐ No

Field work, collection and transport

Field conditions

The fin whale samples used for whole genome resequencing were collected throughout a 22 year time period. Environmental conditions were not relevant for our study and are therefore not reported here.

Location

The locations (latitude and longitude) at which we obtained the samples were:

Eastern North Pacific population:
ENPCA1 33.383333 -119.5
ENPCA2 36.616666 -126.866666
ENPCA3 34.95 -122.133333
ENPCA4 32.866666 -120.5
ENPCA5 33.133333 -117.7
ENPCA6 41.1 -127.5
ENPCA7 39.716666 -124.95
ENPCA8 34.833333 -121.5
ENPCA9 36.626 -122.4107
ENPOR10 45.633333 -128.233333
ENPOR11 44.266666 -128.266666
ENPOR12 45.283333 -126.133333
ENPOR13 44.116666 -126.566666
ENPWA14 47.116666 -127.933333
ENPWA15 47.483333 -126.083333
ENPBC16 52.6 -130.166666

ENPBC17 51.116666 -132
ENPBC18 54.316666 -132.7
ENPAK19 56.833333 -153.216666
ENPAK20 55.983333 -156.85
ENPAK21 59.483333 -149.35
ENPAK22 57.716666 -154.05
ENPAK23 58.8 -140.033333
ENPAK24 59.05 -141.933333
ENPAK25 59.15 -141.116666
ENPAK26 59.65 -142.683333
ENPAK27 59 -144.15
ENPAK28 57.083333 -151.516666
ENPAK29 58.983333 -150.4
ENPAK30 58.533333 -150.133333

Gulf of California population (there are two samples from which we do not have latitude and longitude information):
GOC002 24.3359 -110.5066
GOC006 24.37767 -110.53494
GOC010 24.4587 -110.6120
GOC025 28.9565 -113.3442
GOC038 29.70183 -112.91902
GOC050 25.89263 -111.13428
GOC053 25.5379 -110.9627
GOC063 24.73563 110.11558
GOC068 25.77556 111.1126
GOC071 25.76998 111.1147
GOC077 25.91183 111.10533
GOC080 29.14291 -113.5785
GOC082 29.0186 -113.4773
GOC086 28.9980 -113.3089
GOC091 28.9550 -113.3630
GOC100 NA NA
GOC111 28.6937 -112.2473
GOC112 28.6988 -112.2825
GOC116 NA NA
GOC125 29.3636 -112.5922

Access & import/export

All field work and export of samples from the Gulf of California were obtained under the appropriate collecting permits issued by the from the was approved by the Mexican Wildlife Agency (Dirección General de Vida Silvestre, Subsecretaría de Gestión para la Protección Ambiental, Secretaría del Medio Ambiente y Recursos Naturales; permit numbers: D0070(2)-0598, D00700(2)-14093, D00750-1537 and SGPA/DGVS/-0576). The samples from the Gulf of California were exported to the U.S.A. under Mexican CITES Permit Number: 11526 and U.S. CITES Permit Number: 07US774223/9. Samples from the Eastern North Pacific were collected by the Southwest Fisheries Science Center (California, USA) in accordance with national guidelines and regulations (permit numbers: NMFS-873, NMFS-1026, NMFS-774-1437, NMFS 0782-1438, NMFS-774-1714, NMFS-774-1437, NMFS-14097 and NMFS-19091). The Canadian samples were imported to the U.S.A. under Canadian CITES Permit Number: CA 013 and U.S. CITES Permit Number: 10US690343/9.

Disturbance

The sampling protocol we followed is minimally invasive, it only takes a small skin sample and the animal is not harassed for long periods of time. Therefore, the sampling activities that we performed are very unlikely to have caused any kind of disturbance of this natural system.

Reporting for specific materials, systems and methods

We require information from authors about some types of materials, experimental systems and methods used in many studies. Here, indicate whether each material, system or method listed is relevant to your study. If you are not sure if a list item applies to your research, read the appropriate section before selecting a response.

Materials & experimental systems

n/a	Involved in the study
☒	☐ Antibodies
☒	☐ Eukaryotic cell lines
☒	☐ Palaeontology and archaeology
☐	☒ Animals and other organisms
☒	☐ Clinical data
☒	☐ Dual use research of concern

Methods

n/a	Involved in the study
☒	☐ ChIP-seq
☒	☐ Flow cytometry
☒	☐ MRI-based neuroimaging

Animals and other research organisms

Policy information about studies involving animals; ARRIVE guidelines recommended for reporting animal research, and Sex and Gender in Research

Laboratory animals	This study did not involve laboratory animals
Wild animals	We did not carry out any experiment on wild individuals. Fin whales were not captured for our study, we obtained the small skin biopsy samples remotely through the deployment of a biopsy dart.
Reporting on sex	The sex of fin whales is very difficult to determine in the field. We only determined the sex from around half of the samples we collected. Although the sex of the whales can be determine with molecular approaches, we did not have the funding to do so. However, sex was not relevant for any of the analyses or to fulfill any of our objectives, therefore, it was not reported.
Field-collected samples	In the field, the biopsy sample were immediately preserved in vials containing an ethanol solution at 90% of concentration. Once in the laboratory, the biopsies were stored in freezers at -20°C or -80°C until the DNA was extracted. After extraction, the DNA was stored at -80°C until it was sent for whole-genome resequencing.
Ethics oversight	The field work and sampling protocol was approved by the Mexican Wildlife Agency (Dirección General de Vida Silvestre, Subsecretaría de Gestión para la Protección Ambiental, Secretaría del Medio Ambiente y Recursos Naturales) and the Southwest Fisheries Science Service in the United States.

Note that full information on the approval of the study protocol must also be provided in the manuscript.
